# No Cost Sharing for Public Assistance Recipients and Health Service Usage in Japan

**DOI:** 10.1001/jamahealthforum.2025.3713

**Published:** 2025-10-24

**Authors:** Chihiro Shiota, Kenji Takeuchi, Taro Kusama, Yudai Tamada, Futoshi Oda, Megumi Maeda, Ken Osaka, Haruhisa Fukuda

**Affiliations:** 1 Department of International and Community Oral Health, Tohoku University Graduate School of Dentistry, Miyagi, Japan; 2 Division of Statistics and Data Science, Liaison Center for Innovative Dentistry, Tohoku University Graduate School of Dentistry, Miyagi, Japan; 3 Department of Preventive Medicine, Nagoya University Graduate School of Medicine, Aichi, Japan; 4 Department of Health Care Administration and Management, Kyushu University Graduate School of Medical Sciences, Fukuoka, Japan

## Abstract

**Importance:**

In Japan, the public assistance (PA) system for low-income individuals provides medical aid that covers all health care costs (no cost sharing). However, little is known about how health care service utilization changes after PA certification.

**Objective:**

To examine the trajectories of outpatient medical and dental care utilization among PA recipients before and after PA certification.

**Design, Setting, and Participants:**

Longitudinal data from a municipality in Japan participating in the Longevity Improvement Fair Evidence Study from 2017 to 2022 were used. Generalized estimating equations were used to calculate absolute differences and relative ratios in health care service utilization before and after PA certification. An interrupted time-series analysis (ITSA) was performed to examine the trends in health care service use based on monthly panel data. The elasticity of each health care service was also calculated. The participants were individuals who switched from National Health Insurance to PA and were 20 years and older. The analysis was performed in 2024.

**Exposure:**

PA certification.

**Main Outcomes and Measures:**

Outpatient medical and dental care expenditures, visits, and unit costs per month from 1 year before to 1 year after PA certification.

**Results:**

Overall, 2893 study participants were analyzed (mean [SD] age, 54.2 [15.3] years; 1501 women [51.9%]). For outpatient medical care, the mean (SD) expenditures per month before and after PA certification were ¥16 565 (¥53 159) and ¥22 442 (¥53 443), respectively (converting to US dollars using the rate of ¥100 = $0.68 based on 2025 rates). For outpatient dental care, the mean (SD) expenditures per month before and after PA certification were ¥1727 (¥3726) and ¥3978 (¥6894), respectively. The ITSA showed significant increases in outpatient expenditure immediately after PA certification: ¥2681 (95% CI, 317-5046) and ¥2330 (95% CI, 1896-2764) in medical and dental care, respectively.

**Conclusions and Relevance:**

This study found that eliminating cost sharing under a PA system was associated with increased outpatient health service use, particularly for dental care. These findings highlight financial barriers to care among low-income populations and suggest that no cost-sharing policies can improve access but may require policy measures to address overuse.

## Key Points

**Question:**

Is no cost sharing for health care services for public assistance recipients associated with increased medical and dental care utilization?

**Findings:**

In this retrospective cohort study including 2893 patients certified for public assistance, outpatient care was associated with increased medical and dental settings after switching to no cost sharing. Dental care use showed significantly greater elasticity than medical care use.

**Meaning:**

No cost sharing on health care services for public assistance recipients was associated with higher outpatient medical and dental care utilization, highlighting the importance of monitoring the use of appropriate health care services before and after implementing no cost sharing.

## Teaser

This cohort study examines the association of cost sharing for health care services for public assistance recipients with medical and dental care utilization.

## Introduction

Cost sharing represents a major barrier to receiving essential medical services, particularly for low-income individuals who are more likely to encounter such obstacles.^1^ In addition, although low-income individuals may be considered to have poorer health conditions compared with the general population, low-income groups may be more likely to experience poorer health conditions if they hesitate to access necessary medical services.^2,3^ In Japan, although public assistance (PA) is available to households living below the poverty line, PA recipients tend to have more disadvantageous health and well-being status compared with the general population.^4^ This health inequality could be because the no cost sharing for health services benefit provided for PA recipients has not solved the lack of health service use.

There is a discrepancy in previous studies regarding whether no cost sharing increases health service use among PA recipients compared with medical insurance enrollees. A Japanese cross-sectional study and the Ministry of Health, Labour and Welfare reported that PA recipients use more medical services than other health insurance enrollees among Japanese adults.^5,6^ However, another cross-sectional study demonstrated that PA recipients made fewer dental clinic visits than other health insurance enrollees.^7^ One of the reasons for this discrepancy is that the characteristics and social backgrounds of PA recipients differ from those of the general population.^8^ Other countries have similarly reported mixed health care service use as responses to enrollment in medical aid programs.^9,10,11,12^ Therefore, it is necessary to remove these biases and estimate the causal relationship between no cost sharing and the use of health services by PA recipients.

We hypothesized that PA certification (no cost sharing) increases health care utilization, specifically outpatient medical and dental expenditures, number of visits, and unit costs. To test this hypothesis, this study performed an interrupted time-series analysis (ITSA) of data from before and after PA certification for a single population of PA recipients in Japan to examine the causal effects of no cost sharing on health service use. Specifically, ITSA enables us to disregard the distribution of age and economic status in the population and to exclude the effects of unmeasured confounders.^13,14^ Moreover, we evaluated price elasticities to check whether the trends of each health care use among PA recipients differed between medical and dental care.

## Methods

### Study Design and Data Source

This study was approved by the Kyushu University institutional review board for Clinical Research and ethics committee of Tohoku University Graduate School of Dentistry. Written informed consent was obtained from all participants. This study followed the Strengthening the Reporting of Observational Studies in Epidemiology (STROBE) reporting guidelines.

This retrospective cohort study was based on data from the Longevity Improvement Fair Evidence (LIFE) Study,^15^ a longitudinal community-based database project that collected administrative health care claims data and other health and social data in collaboration with municipal governments. In the LIFE study, a unique identifier was assigned to each participant by data managers, and each data point can be linked at the individual level using the pseudonymized personal identifier. This study used data between April 2017 and March 2022 collected from a municipality that provided the health care claims data of the National Health Insurance (NHI) enrollees, the National Survey on Public Assistance Recipient data, and the Basic Resident Register data. Variability in broader municipality-level characteristics, including health care clinician density and service availability, is likely limited due to the use of a single municipality. Additional information on the Japanese health care system and differences between NHI and PA is provided in eMethods 1 and eTable 1 in Supplement 1. The PA dataset included monthly certification information, with no marked temporal or seasonal fluctuations during the study period. The Basic Resident Register data provides demographic data, including address, sex, and date of birth.

### Participants

Receipt types of health care claims data were recorded based on the individual’s insurance. Eligible participants were individuals who were newly certified for PA between April 2018 and April 2021 and had at least 1 health care claim with NHI receipt type within 1 year prior to PA certification. The municipality we used did not have NHI Register data, and we regarded health care claims with NHI receipt type as NHI enrollment for 1 year before PA certification. This assumption was justified under Japan’s universal health insurance system.^16^ We excluded individuals with missing identification, those younger than 20 years when they were certified for PA, and those who died within 1 year after PA certification. In addition, individuals whose health care records did not correspond to PA receipt types (NHI or the Latter-Stage Older Persons Health Care System) within 1 year after PA certification, or whose data could not be linked with the Basic Resident Register data, were excluded from the analysis (eFigure 1 in Supplement 1).

### Outcome Variables

The primary outcomes were monthly health care expenditures, number of visits, and unit costs for medical and dental care before and after PA certification. Claims data were structured by month, medical institution, and claim type (ie, outpatient, inpatient, pharmacy, and diagnosis procedure combination hospitals), with each row representing a unique combination. For instance, 3 separate records would appear when a patient visited 3 outpatient clinics within a single month. Consistent with previous literature, data from the PA certification month and the immediately preceding month were excluded from analyses to account for temporal instability around the intervention (donut-hole design).^17,18,19,20,21,22,23,24^ The mean monthly outpatient medical and dental expenditures, as well as the number of visits, were calculated for the 11 months before and the 11 months after the month of PA certification. For the number of visits, each row of data included data on the number of days per medical institution and receipt types, which were used to calculate the total number of outpatient visits per month for medical and dental care. Moreover, we computed unit costs by dividing monthly outpatient medical and dental expenditures by the number of visits per month.

### Exposure Variable

The exposure of interest was the transition from NHI enrollment to PA certification. The PA system began in 1945, with recipients increasing to approximately 2 million in 2022.^25^ PA eligibility requires meeting strict criteria, such as a lack of financial assets (eg, house, car). The local PA office assesses PA certification in response to each application. PA recipients can receive monthly minimum income benefits and are fully exempt from paying for medical and dental care. The month of PA certification was available in Public Assistance Recipient data and defined as month 0, referring to the month in which PA certification and enrollment occurred. The 12 months prior were identified as months −12 to −1, whereas the 12 months following certification were defined as months 0 to 11.

### Statistical Analysis

Generalized estimating equation (GEE) models with γ family and identity and log link functions were used to estimate the absolute differences and relative ratios for monthly medical and dental expenditures, number of visits, and unit costs before and after PA certification. In addition, the xtset, xtgee, and xtitsa commands in Stata statistical software were used, with the GEE and ITSA model formulas presented in eMethods 2 in Supplement 1. These models were applied to assess the temporal trends and level changes in monthly outpatient medical and dental expenditures, number of visits, and unit costs. Sex (male/female), age group (20-29, 30-39, 40-49, 50-59, 60-69, or 70-75 years) at cohort entry month (ie, the month of PA certification, month 0), and the year of PA certification were adjusted. Three sensitivity analyses were conducted to assess the robustness of the findings (eMethods 3 in Supplement 1).

To evaluate the differences in health care utilization between medical and dental care, the price elasticities for each type of medical and dental expenditures, number of visits, and unit costs were calculated (eMethods 4 in Supplement 1). All statistical analyses were performed using Stata statistical software (version 17.0, Stata Corp). Analysis took place in 2024.

## Results

### Study Population

A total of 2893 participants were included in the analysis (eFigure 2 in Supplement 1). The mean (SD) age at PA certification was 54.2 (15.3) years, and 1501 (51.9%) were women (eTable 2 in Supplement 1).

### Changes in Outcomes Before and After Public Assistance Certification and Price Elasticities

Descriptive statistics for outpatient medical and dental care before and after PA certification are presented in Table 1. The mean (SD) monthly outpatient medical expenditures increased from ¥16 565 (¥53 159) before PA certification to ¥22 442 (¥53 443) after (¥100 = $0.68 based on 2025 rates). For outpatient dental care, the mean (SD) monthly expenditure increased from ¥1727 (¥3726) to ¥3978 (¥6894). All calculated price elasticities were less than 1, indicating inelastic demand across all categories.

**Table 1.** Descriptive Statistics of Outpatient Medical and Dental Care Use Among the Analytic Sample of 2893 Individuals^a^

Variable	Public assistance certification, mean (SD)		Price elasticity
	Before	After	
**Medical care**			
Expenditures, ¥	16 565 (53 159)	22 442 (53 443)	−0.15
Visits, No.	1.54 (2.11)	2.07 (2.48)	−0.15
Unit costs, ¥	6450 (33 890)	8428 (31 624)	−0.13
**Dental care**			
Expenditures, ¥	1727 (3726)	3978 (6894)	−0.39
Visits, No.	0.21 (0.44)	0.41 (0.66)	−0.32
Unit costs, ¥	843 (1565)	1747 (2677)	−0.35

^a^ For currency conversion, ¥100 was considered equivalent to $0.68 based on 2025 rates.

### Absolute Differences and Relative Ratios of Outcomes Before and After Public Assistance Certification

In the adjusted model (model 2), outpatient medical expenditures increased by ¥4942 (95% CI, 3301-6584; Table 2), whereas dental expenditures increased by ¥2249 (95% CI, 1955-2543) after PA certification. Similar increases were observed in the number of visits and unit costs.

**Table 2.** Absolute Differences and Relative Ratios of Outpatient Medical/Dental Care Use Among the Analytic Sample of 2893 Individuals^a^^,^^b^

Variable	Absolute difference (95% CI)			
	Medical care		Dental care	
	Crude model	Adjusted model^c^	Crude model	Adjusted model^c^
Expenditures, ¥	5878 (3989-7767)	4942 (3301-6584)	2251 (1955-2546)	2249 (1955-2543)
Visits, No.	0.53 (0.43-0.62)	0.43 (0.34-0.52)	0.20 (0.17-0.22)	0.20 (0.17-0.22)
Unit costs, ¥	1978 (1205-2752)	1898 (1282-2513)	904 (795-1013)	906 (797-1014)
	RR (95% CI)	RR (95% CI)^c^	RR (95% CI)	RR (95% CI)^c^
Expenditures	1.35 (1.23-1.49)	1.35 (1.23-1.47)	2.30 (2.10-2.53)	2.31 (2.10-2.54)
Visits, No.	1.34 (1.27-1.42)	1.32 (1.26-1.40)	1.92 (1.77-2.10)	1.93 (1.77-2.10)
Unit costs	1.31 (1.19-1.44)	1.31 (1.21-1.42)	2.07 (1.92-2.24)	2.08 (1.93-2.25)

Abbreviation: RR, relative ratio.

^a^ For currency conversion, ¥100 was considered equivalent to $0.68 based on 2025 rates.

^b^ Expenditures, visits, and unit costs are shown as means per month.

^c^ Adjusted for age and sex in the month the participants were certified for public assistance and the year the participants were certified for public assistance.

### Trend Trajectories in Outcomes Before and After Public Assistance Certification

**Figure 1.**
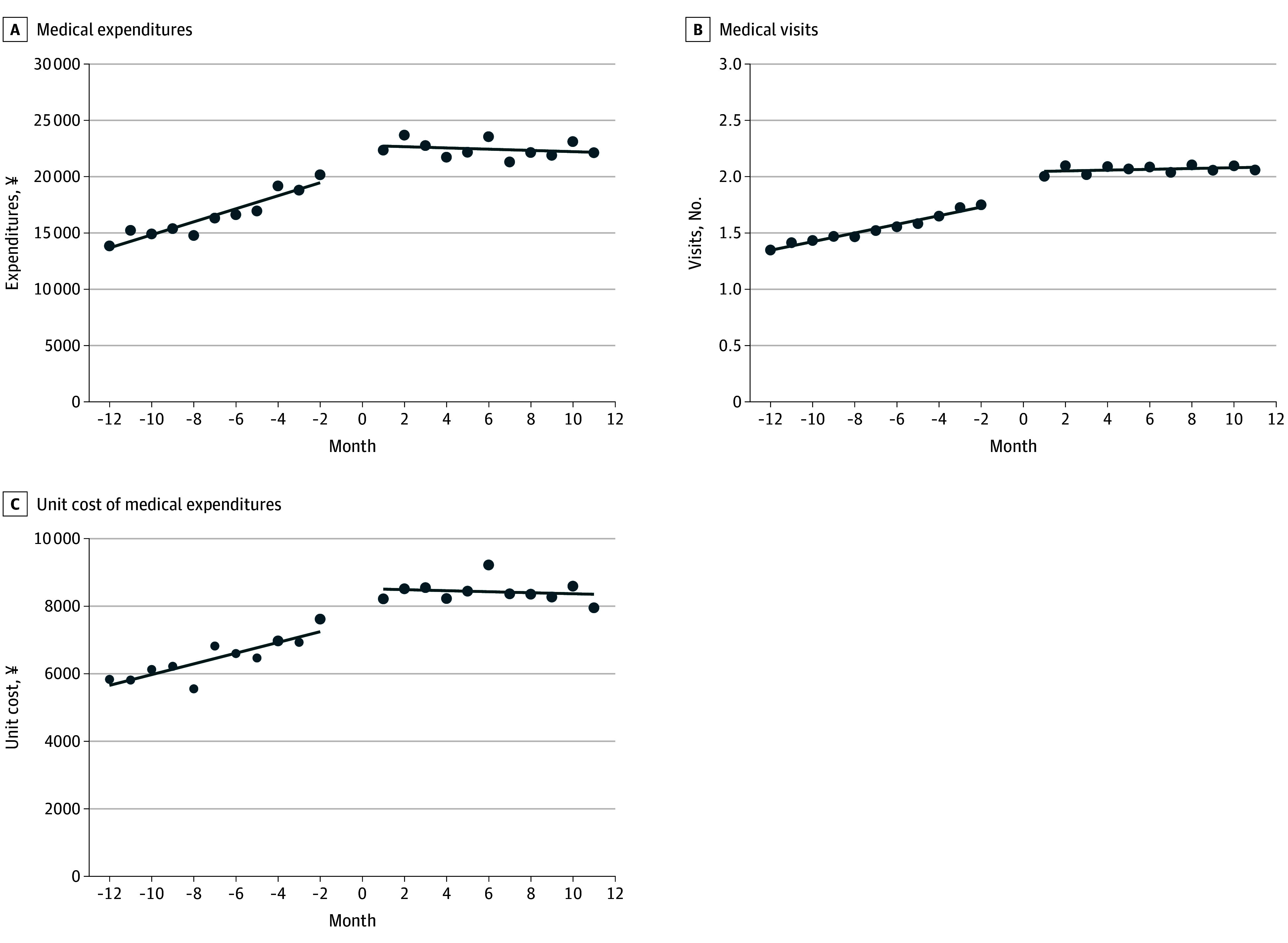
Interrupted Time-Series Analysis of Outpatient Medical Care Use Among the Analytic Sample of 2893 Individuals A, Level change, ¥2681 (95% CI, 317-5046); trend before public assistance certification, ¥579 (95% CI, 373-784); trend after public assistance certification, −55 (95% CI, −297 to 187). B, Level change, 0.26 (95% CI, 0.14-0.37) times; trend before public assistance certification, 0.04 (95% CI, 0.03-0.05) times; trend after public assistance certification, 0.002 (95% CI, −0.01 to 0.01) times. C, Level change, ¥1227 (95% CI, 457-1997); trend before public assistance certification, ¥162 (95% CI, 91–232); trend after public assistance certification, −7.8 (95% CI, −87 to 71). The month of the PA certification was defined as month 0. The 12 months prior were identified as months −12 to −1, whereas the 12 months following the PA certification were defined as months 0 to 11. For currency conversion, ¥100 was considered equivalent to $0.68 based on 2025 rates. PA indicates public assistance.

**Figure 2.**
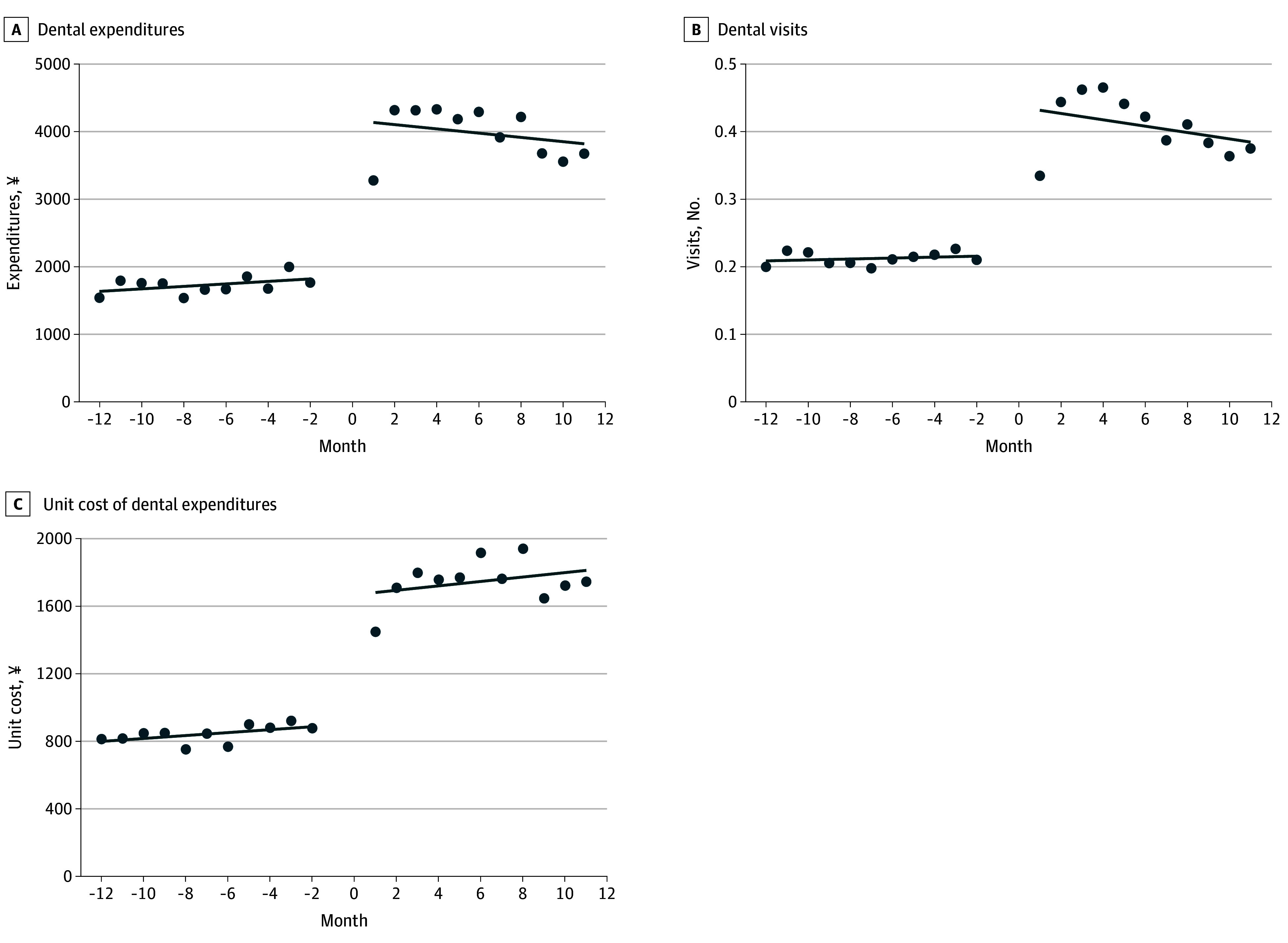
Interrupted Time-Series Analysis of Outpatient Dental Care Use Among the Analytic Sample of 2893 Individuals A, Level change, ¥2330 (95% CI, 1896-2764); trend before public assistance certification, ¥18 (95% CI, −15 to 51); trend after public assistance certification, −365 (95% CI, −91 to 22). B, Level change, 0.22 (95% CI, 0.18-0.26) times; trend before public assistance certification, 0.001 (95% CI, −0.003 to 0.004) times; trend after public assistance certification, −0.01 (95% CI, −0.01 to 0.0003) times. C, Level change, 794 (95% CI, 643–945); trend before public assistance certification, ¥6.5 (95% CI, −6.3 to 19); trend after public assistance certification, ¥16 (95% CI, 4.8-36). The month of the PA certification was defined as month 0. The 12 months prior were identified as months −12 to −1, whereas the 12 months following the PA certification were defined as months 0 to 11. For currency conversion, ¥100 was considered equivalent to $0.68 based on 2025 rates. PA indicates public assistance.

The ITSA indicated significant level changes in all outcome variables following PA certification (Table 3, Figure 1 and Figure 2). Immediate postcertification increases in monthly medical and dental expenditures were observed (¥2681 [95% CI, 317-5046] and ¥2330 [95% CI, 1896-2764], respectively). For the monthly number of outpatient visits, immediate postcertification increases were observed for medical care (0.26 [95% CI, 0.14-0.37]) and dental care (0.22 [95% CI, 0.18-0.26]). Immediate postcertification increases in monthly outpatient unit costs for medical care (¥1227 [95% CI, 457-1997]) and dental care (¥794 [95% CI, 643-945]) were also observed (Table 3).

**Table 3.** Interrupted Time-Series Analysis of Medical and Dental Care Use Among the Analytic Sample of 2893 Individuals^a^^,^^b^

Variable	Level change^c^ (95% CI)	Trend before public assistance certification (95% CI)	Trend after public assistance certification (95% CI)
**Medical care**			
Expenditures, ¥^d^	2681 (317 to 5046)	579 (373 to 784)	−55 (−297 to 187)
Visits, No.	0.26 (0.14 to 0.37)	0.04 (0.03 to 0.05)	0.002 (−0.01 to 0.01)
Unit costs, ¥	1227 (457 to 1997)	162 (91 to 232)	−7.8 (−87 to 71)
**Dental care**			
Expenditures, ¥	2330 (1896 to 2764)	18 (−15 to 51)	−35 (−91 to 22)
Visits, No.	0.22 (0.18 to 0.26)	0.001 (−0.003 to 0.004)	−0.01 (−0.01 to −0.0003)
Unit costs, ¥	794 (643 to 945)	6.5 (−6.3 to 19)	16 (4.8 to 36)

^a^ For currency conversion, ¥100 was considered equivalent to $0.68 based on 2025 rates.

^b^ Expenditures, visits, and unit costs are shown as averages per month. Adjusted for age and sex in the month the participants were certified for public assistance and the year the participants were certified for public assistance.

^c^ Level change refers to the amount of change in the intercept of the outcome from immediately before to immediately after public assistance certification.

^d^ Only medical expenditure is calculated using the option “family (gaussian)” to converge.

Precertification trends across outcomes showed gradual increases approaching month 0. After PA certification, all outcomes increased sharply, followed by a general decline. Exceptions included a modest upward trend in outpatient medical visits and a sustained increase in dental unit costs (Figure 1 and Figure 2).

### Sensitivity Analyses

The results of 3 sensitivity analyses are provided in eResults 1 in Supplement 1. All findings were consistent with the main analysis.

## Discussion

### Summary of Main Findings

In this prospective cohort study, the elimination of cost sharing was associated with an increase in the use of outpatient health services—as measured by monthly expenditures, number of visits, and unit costs for both medical and dental care—among PA recipients in Japan. The estimated price elasticities for expenditures, visits, and unit costs were −0.15, −0.15, and −0.13, respectively, for medical care and −0.39, −0.32, and −0.35, respectively, for dental care. All estimates were below 1, indicating an inelastic demand, although dental services demonstrated greater elasticity than medical services.

### Comparison with Previous Studies

Our findings on outpatient medical care are consistent with those of previous cross-sectional research, which reported that PA certification increased monthly medical expenditures by 17.5% to 22.9% and the monthly number of visits by 23.1% to 27.8%.^5^ The national average of 2.5 outpatient visits per month among PA recipients^6^ is similar to our observation of 2.07 visits. Meanwhile, the mean medical expenditure in our cohort was higher (¥22 422) than previously reported; this difference may be due to 2 factors. First, the target participants were individuals with claims data that included an NHI receipt type at least once before PA certification, potentially reflecting individuals who use more health care services than general NHI enrollees. However, according to the 2022 national report, NHI enrollees utilize health care services 8 to 11 times per year, and are likely to access health care services at least once per year,^26^ suggesting a limited risk of selection bias. Second, the study design focused on the immediate period before and after PA certification—a timeframe responsiveness to certification may be elevated.

The GEE models in our study showed larger increases: 35% and 32% in medical expenditures and number of visits, respectively. The ITSA results supported these findings, with level changes of ¥2681 in expenditures and 0.26 in visits, consistent with the findings of prior regression discontinuity studies that examined similar cost-sharing reductions.^17,19^

To our knowledge, this is the first study to quantify outpatient unit costs before and after PA certification. The average unit cost increased by ¥1898 (1.31-fold) postcertification in the GEE models, and a level change of ¥1227 was observed in the ITSA models. This likely reflected a combined increase in expenditure and visit frequency.

Similarly, in terms of dental care, our results exhibited significant increases in postcertification expenditures, number of visits, and unit costs. Although previous studies have investigated the effects of dental insurance expansion,^27,28,29,30^ few have examined dental service utilization under the no cost-sharing benefit in Japan. Notably, we observed a 92% increase in dental visits, substantially greater than estimates from US-based simulations (eg, 0.37 additional visits).^30^ This disparity may be due to differences in the health care financing systems. In Japan, nearly all citizens are enrolled in public insurance with a 30% copayment rate, and PA recipients fully receive health care services if the services are covered by public insurance.^16^ This accessibility may lead to more pronounced changes in utilization following the PA certification.

For price elasticities, previous studies reported that the elasticities of outpatient medical care ranged from approximately 0 to −0.16,^17,18,19,31^ whereas those of dental care ranged from near 0 to −0.47.^29,32,33,34,35^ Our estimates were consistent with these findings and further supported the notion that dental care utilization is more price-sensitive than medical care.^36^

### Robustness to Potential Reverse Causality and Care Type

To address concerns regarding reverse causality—where deteriorating health reduces income, and increases enrollment in PAs with no cost sharing, we conducted a sensitivity analysis excluding individuals who reported initiating PA due to chronic illness or disease. The findings remained consistent, underscoring the impact of reduced cost sharing on health care utilization. When emergency department users were excluded from the analysis, the results remained consistent, suggesting that increases in utilization extended beyond frequent or acute care users. These findings suggest that the increases in outpatient health service utilization were not attributable to specific subpopulations.

### Interpretation and Policy Implications

Our findings indicate that PA recipients may delay necessary care owing to costs, particularly dental services, due to financial barriers, opting to wait until they are eligible for no-cost care. Dental care is more likely to be deferred than medical care, especially among low-income individuals, who may seek treatment only when problems become severe.^24^

Increased health care utilization following reduced cost sharing may improve health outcomes, including activities of daily living, mental health, and even mortality.^37,38,39,40^ A Japanese study^18^ found that reducing copayment rates from 30% to 10% improved self-rated health status among low-income older adults. Although some studies reported no significant expenditure changes for childhood dental care and sicker older adults,^19,41^ our findings imply that the complete elimination of cost sharing (30% to 0%) can substantially increase outpatient utilization and potentially improve access to care.

However, increased utilization does not necessarily equate to improved health status. Overuse, potentially driven by moral hazard or supplier-induced demand, is a concern in no cost-sharing systems.^5,37,42,43,44,45,46^ To prevent excessive billing and inappropriate care, the government monitors excessive medical use and revokes a medical institution’s designation. Some municipalities have implemented proactive oversight measures, including claims data from welfare offices, to identify duplicate prescriptions, which has improved approximately half of such cases.^47^ However, these practices are not uniformly adopted, and empirical evidence on the effectiveness of monitoring in reducing supplier-induced demand remains limited. From a health policy perspective, introducing minimal cost sharing (eg, 1%) for excessive users, regardless of health status, may be effective.^48^

### Strengths and Limitations

This study has some limitations. First, the study used data from a single municipality, which may limit the generalizability of our findings. However, a similar age distribution was observed in a 2022 national PA survey, with a slightly higher proportion of older age groups than all PA recipients in the current study (eTable 12 in Supplement 1).^25^ Second, we lacked data on continued PA enrollment or dropout. However, national data show that the average PA enrollment duration exceeds 9 years, with fewer than a quarter of recipients dropping out within 1 year.^25^ Thus, withdrawal during our 1-year follow-up is likely limited and unlikely to have biased the results. Moreover, after excluding individuals without health care claims after PA certification (potential withdrawals), we observed similar increases in outpatient medical and dental service utilization. Third, only sex, age, and the year of PA certification were available as covariates. Nonetheless, sensitivity analyses showed similar trends, suggesting robustness. Fourth, requiring individuals to have NHI claims before PA certification may introduce selection bias. However, because NHI enrollees typically make at least 1 medical visit annually,^26^ this bias is likely minimal. Fifth, we assessed unit costs and expenditures uniformly, though they may differ by care type. Excluding emergency department users in the sensitivity analysis yielded similar results; future research should explore service-specific differences.

This cohort study also had some strengths. To our knowledge, this is the first longitudinal study evaluating within-individual changes in both medical and dental outpatient care before and after PA certification. The use of ITSA helped account for unmeasured confounding. Simultaneously assessing expenditures, visits, and unit costs provides a comprehensive view of health care utilization.

## Conclusions

This study found that the elimination of cost sharing under Japan’s PA system was associated with substantial increases in outpatient medical and dental care utilization, as evidenced by higher expenditures, more frequent visits, and elevated unit costs. Despite these increases, all measures exhibited inelastic demand, particularly for dental care, which showed greater price sensitivity. These findings suggest that financial barriers often delay care for low-income individuals, particularly dental care services. Although increased utilization may improve health outcomes, the potential for overuse underscores the need for policies such as targeted cost sharing or utilization monitoring.

## Notes

Supplement 1. eTable 1. Comparison of National Healthcare Insurance and Public Assistance System eTable 2. Descriptive Characteristics of the Analytic Sample (n = 2,893) eTable 3. Descriptive Characteristics of the Analytic Sample in the Sensitivity Analysis, Excluding Those with Zero Healthcare Expenditures After Public Assistance Certification (n = 2,821) eTable 4. Descriptive Statistics of Outpatient Medical and Dental Care Use in the Sensitivity Analysis, Excluding Those with Zero Healthcare Expenditures After Public Assistance Certification (n = 2,821) eTable 5. Absolute Differences and Relative Ratios of Outpatient Medical and Dental Care Use in the Sensitivity Analysis, Excluding Those with Zero Healthcare Expenditures After Public Assistance Certification (n = 2,821) eTable 6. Interrupted-Time Series Analysis of Medical and Dental Care Use in the Sensitivity Analysis, Excluding Those Who Reported Initiating Public Assistance Due to Chronic Illness or Disease (n = 2,821) eTable 7. Absolute Differences and Relative Ratios of Outpatient Medical and Dental Care Use in the Sensitivity Analysis, Excluding Those Who Reported Initiating Public Assistance Due to Chronic Illness or Disease (n = 2,308) eTable 8. Interrupted-Time Series Analysis of Medical and Dental Care Use in the Sensitivity Analysis, Excluding Those Who Reported Initiating Public Assistance Due to Chronic Illness or Disease (n = 2,308) eTable 9. Descriptive Statistics of Outpatient Medical and Dental Care Use in the Sensitivity Analysis, Excluding Those Who Used the Emergency Department (n = 2,691) eTable 10. Absolute Differences and Relative Ratios of Outpatient Medical and Dental Care Use in the Sensitivity Analysis, Excluding Those Who Used the Emergency Department (n = 2,691) eTable 11. Interrupted-Time Series Analysis of Medical and Dental Care Use in the Sensitivity Analysis, Excluding Those Who Used the Emergency Department (n = 2,691) eTable 12. Comparison of Sex and Age Distribution Between the Target Population in This study and the Public Assistance Recipients Based on National data eFigure1. Design of the Analytic Sample (n = 2,893) eFigure 2. Participant Flow for the Analytic Sample (n = 2,893) eFigure 3. Design of the Analytic Sample in the Sensitivity Analysis, Excluding Those Whose Healthcare Expenditures were Zero After Public Assistance Certification (n = 2,821) eFigure 4. Participant Flow for the Analytic Sample in the Sensitivity Analysis, Excluding Those with Zero Healthcare Expenditures After Public Assistance Certification (n = 2,821) eFigure 5. Interrupted Time-Series Analysis of Outpatient Medical Care Use in the Sensitivity Analysis, Excluding Those Who Reported Initiating Public Assistance Due to Chronic Illness or Disease (n = 2,821) eFigure 6. Interrupted Time-Series Analysis of Outpatient Dental Care Use in the Sensitivity Analysis, Excluding Those Who Reported Initiating Public Assistance Due to Chronic Illness or Disease (n = 2,821) eFigure 7. Interrupted Time-Series Analysis of Outpatient Medical Care Use in the Sensitivity Analysis, Excluding Those Who Reported Initiating Public Assistance Due to Chronic Illness or Disease (n = 2,308) eFigure 8. Interrupted Time-Series Analysis of Outpatient Dental Care Use in the Sensitivity Analysis, Excluding Those Who Reported Initiating Public Assistance Due to Chronic Illness or Disease (n = 2,308) eFigure 9. Interrupted Time-Series Analysis of Outpatient Medical Care Use in the Sensitivity Analysis, Excluding Those Who Used the Emergency Department (n = 2,691) eFigure 10. Interrupted Time-Series Analysis of Outpatient Dental Care Use in the Sensitivity Analysis, Excluding Those Who Used the Emergency Department (n = 2,691) eMethods 1. Introduction to the Japanese Healthcare System eMethods 2. Formulas in Generalized Estimating Equation Models and Interrupted Time-Series Analysis Models Used in This Study eMethods 3. Overview of Three Sensitivity Analyses eMethods 4. Formula for Calculating Price Elasticities of Expenditure, Number of Visits, and Unit Cost eResults1: Results of Three Sensitivity Analyses

Supplement 2. Data Sharing Statement
