# Supplementary material for: No Cost Sharing for Public Assistance Recipients and Health Service Usage in Japan
Source: JAMA Health Forum. 2025 Oct 24;6(10):e253713. doi: 10.1001/jamahealthforum.2025.3713 (PMC12552928; doi:10.1001/jamahealthforum.2025.3713)
Supplement: Supplement 1. — eTable 1. Comparison of National Healthcare Insurance and Public Assistance System eTable 2. Descriptive Characteristics of the Analytic Sample (n = 2,893) eTable 3. Descriptive Characteristics of the Analytic Sample in the Sensitivity Analysis, Excluding Those with Zero Healthcare Expenditures After Public Assistance Certification (n = 2,821) eTable 4. Descriptive Statistics of Outpatient Medical and Dental Care Use in the Sensitivity Analysis, Excluding Those with Zero Healthcare Expenditures After Public Assistance Certification (n = 2,821) eTable 5. Absolute Differences and Relative Ratios of Outpatient Medical and Dental Care Use in the Sensitivity Analysis, Excluding Those with Zero Healthcare Expenditures After Public Assistance Certification (n = 2,821) eTable 6. Interrupted-Time Series Analysis of Medical and Dental Care Use in the Sensitivity Analysis, Excluding Those Who Reported Initiating Public Assistance Due to Chronic Illness or Disease (n = 2,821) eTable 7. Absolute Differences and Relative Ratios of Outpatient Medical and Dental Care Use in the Sensitivity Analysis, Excluding Those Who Reported Initiating Public Assistance Due to Chronic Illness or Disease (n = 2,308) eTable 8. Interrupted-Time Series Analysis of Medical and Dental Care Use in the Sensitivity Analysis, Excluding Those Who Reported Initiating Public Assistance Due to Chronic Illness or Disease (n = 2,308) eTable 9. Descriptive Statistics of Outpatient Medical and Dental Care Use in the Sensitivity Analysis, Excluding Those Who Used the Emergency Department (n = 2,691) eTable 10. Absolute Differences and Relative Ratios of Outpatient Medical and Dental Care Use in the Sensitivity Analysis, Excluding Those Who Used the Emergency Department (n = 2,691) eTable 11. Interrupted-Time Series Analysis of Medical and Dental Care Use in the Sensitivity Analysis, Excluding Those Who Used the Emergency Department (n = 2,691) eTable 12. Comparison of Sex and Age Distribution Between the Target Po [file jamahealthforum-e253713-s001.pdf]

Supplemental Online Content

Shiota C, Takeuchi K, Kusama T, et al. No cost sharing for public assistance recipients and health service usage in Japan. *JAMA Health Forum*. 2025;6(10):e253713. doi:10.1001/jamahealthforum.2025.3713

eTable 1. Comparison of National Healthcare Insurance and Public Assistance System

eTable 2. Descriptive Characteristics of the Analytic Sample (n = 2,893)

eTable 3. Descriptive Characteristics of the Analytic Sample in the Sensitivity Analysis, Excluding Those with Zero Healthcare Expenditures After Public Assistance Certification (n = 2,821)

eTable 4. Descriptive Statistics of Outpatient Medical and Dental Care Use in the Sensitivity Analysis, Excluding Those with Zero Healthcare Expenditures After Public Assistance Certification (n = 2,821)

eTable 5. Absolute Differences and Relative Ratios of Outpatient Medical and Dental Care Use in the Sensitivity Analysis, Excluding Those with Zero Healthcare Expenditures After Public Assistance Certification (n = 2,821)

eTable 6. Interrupted-Time Series Analysis of Medical and Dental Care Use in the Sensitivity Analysis, Excluding Those Who Reported Initiating Public Assistance Due to Chronic Illness or Disease (n = 2,821)

eTable 7. Absolute Differences and Relative Ratios of Outpatient Medical and Dental Care Use in the Sensitivity Analysis, Excluding Those Who Reported Initiating Public Assistance Due to Chronic Illness or Disease (n = 2,308)

eTable 8. Interrupted-Time Series Analysis of Medical and Dental Care Use in the Sensitivity Analysis, Excluding Those Who Reported Initiating Public Assistance Due to Chronic Illness or Disease (n = 2,308)

eTable 9. Descriptive Statistics of Outpatient Medical and Dental Care Use in the Sensitivity Analysis, Excluding Those Who Used the Emergency Department (n = 2,691)

eTable 10. Absolute Differences and Relative Ratios of Outpatient Medical and Dental Care Use in the Sensitivity Analysis, Excluding Those Who Used the Emergency Department (n = 2,691)

eTable 11. Interrupted-Time Series Analysis of Medical and Dental Care Use in the Sensitivity Analysis, Excluding Those Who Used the Emergency Department (n = 2,691)

eTable 12. Comparison of Sex and Age Distribution Between the Target Population in This study and the Public Assistance Recipients Based on National data

eFigure1. Design of the Analytic Sample (n = 2,893)

eFigure 2. Participant Flow for the Analytic Sample (n = 2,893)

eFigure 3. Design of the Analytic Sample in the Sensitivity Analysis, Excluding Those Whose Healthcare Expenditures were Zero After Public Assistance Certification (n = 2,821)

eFigure 4. Participant Flow for the Analytic Sample in the Sensitivity Analysis, Excluding Those with Zero Healthcare Expenditures After Public Assistance Certification (n = 2,821)

eFigure 5. Interrupted Time-Series Analysis of Outpatient Medical Care Use in the Sensitivity Analysis, Excluding Those Who Reported Initiating Public Assistance Due to Chronic Illness or Disease (n = 2,821)

eFigure 6. Interrupted Time-Series Analysis of Outpatient Dental Care Use in the Sensitivity Analysis, Excluding Those Who Reported Initiating Public Assistance Due to Chronic Illness or Disease (n = 2,821)

eFigure 7. Interrupted Time-Series Analysis of Outpatient Medical Care Use in the Sensitivity Analysis, Excluding Those Who Reported Initiating Public Assistance Due to Chronic Illness or Disease (n = 2,308)

eFigure 8. Interrupted Time-Series Analysis of Outpatient Dental Care Use in the Sensitivity Analysis, Excluding Those Who Reported Initiating Public Assistance Due to Chronic Illness or Disease (n = 2,308)

eFigure 9. Interrupted Time-Series Analysis of Outpatient Medical Care Use in the Sensitivity Analysis, Excluding Those Who Used the Emergency Department (n = 2,691)

eFigure 10. Interrupted Time-Series Analysis of Outpatient Dental Care Use in the Sensitivity Analysis, Excluding Those Who Used the Emergency Department (n = 2,691)

eMethods 1. Introduction to the Japanese Healthcare System

eMethods 2. Formulas in Generalized Estimating Equation Models and Interrupted Time-Series Analysis Models Used in This Study

eMethods 3. Overview of Three Sensitivity Analyses

eMethods 4. Formula for Calculating Price Elasticities of Expenditure, Number of Visits, and Unit Cost

eResults1: Results of Three Sensitivity Analyses

This supplemental material has been provided by the authors to give readers additional information about their work.

© 2025 Shiota C et al. *JAMA Health Forum.*

eTable 1. Comparison of National Healthcare Insurance and Public Assistance System

	National Health Insurance (NHI)	Public Assistance (PA)
**Percentage of Total Population**	21.5%.[1]	1.6%[2]
**Coverage Framework**	All treatments covered by public insurance<br>(excluding preventive care)	All treatments covered by public insurance<br>(excluding preventive care)
**Financing Mechanism**	NHI enrollees pay a premium and a 30% out-of-pocket payment<br>(70% are paid by insurance)	No premium and 0% out-of-pocket payment (All are paid by public expense)<br><br>“Iryo-ken” (Tickets to visit hospitals are issued when necessary by each municipality welfare office)
**Eligibility Criteria**	Self-employed or not employed, including part-time workers and retired, and younger than 75 years	Households living below the poverty line without any assets (house, car, etc.), and all ages
**Provider Participation**	No	Yes<br>(Hospital that recipients can visit are defined by municipality welfare office.)<br>(Prescription genetic drugs are recommended.)

© 2025 Shiota C et al. *JAMA Health Forum.*

eTable 2. Descriptive Characteristics of the Analytic sample (n = 2,893)

	n	%
All participants (n = 2,893)		
**Sex**		
**Male**	1,391	48.1
**Female**	1,502	51.9
**Age**		
**20–29**	267	9.2
**30–39**	307	10.6
**40–49**	458	15.8
**50–59**	582	20.1
**60–69**	717	24.8
**70–75**	562	19.4
	**Mean**	**SD**
	54.2	15.3

Abbreviations: SD = Standard deviation.

© 2025 Shiota C et al. *JAMA Health Forum.*

eTable 3. Descriptive Characteristics of the Analytic Sample in the Sensitivity Analysis, Excluding Those with Zero Healthcare Expenditures After Public Assistance Certification (n = 2,821)

	n	%
All participants (n = 2,821)		
**Sex**		
**Male**	1,346	47.7
**Female**	1,475	52.3
**Age**		
**20–29**	261	9.3
**30–39**	298	10.6
**40–49**	441	15.6
**50–59**	565	20
**60–69**	698	24.7
**70–75**	558	19.8
	**Mean**	**SD**
	54.3	15.4

Abbreviations: SD = Standard deviation.

© 2025 Shiota C et al. *JAMA Health Forum.*

eTable 4. Descriptive Statistics of Outpatient Medical and Dental Care Use in the Sensitivity Analysis, Excluding Those with Zero Healthcare Expenditures After Public Assistance Certification (n = 2,821)

		Public assistance certification				
		Before		After		Price elasticity
		Mean	SD	Mean	SD	
**Medical care**	**Expenditures**	16,740	53,615	23,007	54,002	-0.16
	**Visits**	1.56	2.13	2.12	2.49	-0.15
	**Unit costs**	10,228	33,729	11,770	32,327	-0.07
**Dental care**	**Expenditures**	1,740	3,741	4,075	6,950	-0.37
	**Visits**	0.21	0.44	0.42	0.67	-0.33
	**Unit costs**	3,213	4,904	5,007	6,096	-0.22

Abbreviations: SD, standard deviation; JPY, Japanese Yen.

© 2025 Shiota C et al. *JAMA Health Forum.*

eTable 5. Absolute Differences and Relative Ratios of Outpatient Medical and Dental Care Use in the Sensitivity Analysis, Excluding Those with Zero Healthcare Expenditures After Public Assistance Certification (n = 2,821)

	Medical care		Dental care	
	**Crude model**	**Adjusted model**	**Crude model**	**Adjusted model**
	**AD (95% CI)**	**AD (95% CI) [a]**	**AD (95% CI)**	**AD (95% CI) [a]**
**Expenditures (JPY)**	6,267 (4,323–8,281)	5,318 (3,623–7,013)	2,335 (2,032–2,638)	2,331 (2,029–2,633)
**Visits (times)**	0.56 (0.46–0.66)	0.47 (0.38–0.56)	0.20 (0.18–0.23)	0.20 (0.17–0.23)
**Unit costs (JPY)**	1,542 (762–2,323)	1,424 (769–2,079)	1,794 (1,516–2,071)	1,791 (1,513–2,068)
	**RR (95% CI)**	**RR (95% CI) [a]**	**RR (95% CI)**	**RR (95% CI) [a]**
**Expenditures**	1.37 (1.25–1.51)	1.37 (1.25–1.49)	2.34 (2.13–2.58)	2.34 (2.13–2.58)
**Visits**	1.36 (1.29–1.43)	1.34 (1.27–1.42)	1.96 (1.80–2.14)	1.96 (1.80–2.14)
**Unit costs**	1.15 (1.07–1.23)	1.15 (1.08–1.22)	1.56 (1.46–1.66)	1.56 (1.46–1.66)

Abbreviations: AD, absolute difference; RR, relative ratio; CI, Confidence Interval; JPY, Japanese Yen.
[a] Adjusted for age and sex in the month the participants were certified for public assistance and the year when the participants were certified for public assistance.
Expenditures, visits, and unit costs are shown as averages per month.

© 2025 Shiota C et al. *JAMA Health Forum.*

eTable 6. Interrupted-Time Series Analysis of Medical and Dental Care Use in the Sensitivity Analysis, Excluding Those with Zero Healthcare Expenditures After Public Assistance Certification (n = 2,821)

Outcome variables	Level change (95%CI)	Trend before public assistance certification (95%CI)	Trend after public assistance certification (95%CI)
**Medical care**			
**Expenditures (JPY)**	3,217 (865–5,569)	555 (356–754)	-56 (-304–193)
**Visits (times)**	0.31 (0.19–0.43)	0.04 (0.03–0.05)	0.004 (-0.009–0.016)
**Unit cost (JPY)**	1,320 (525–2,115)	149 (72–227)	-15 (-86–56)
**Dental care**			
**Expenditures (JPY)**	2,360 (1,920–2,800)	24 (-8.6–57)	-34 (-93–25)
**Visits (times)**	0.22 (0.18–0.26)	0.001 (-0.002–0.005)	-0.005 (-0.01–0.0002)
**Unit cost (JPY)**	810 (654–965)	11 (-2.0–24)	13 (-8.2–34)

Abbreviations: CI, Confidence Interval; JPY, Japanese Yen.

This analysis is calculated using the option “family(gaussian)” to converge.
Adjusted for age and sex in the month the participants were certified for public assistance and the year when the participants were certified for public assistance.
Level change refers to the amount of change in the intercept of the outcome from immediately before to immediately after public assistance certification.
Expenditures, visits, and unit costs are shown as averages per month.

© 2025 Shiota C et al. *JAMA Health Forum.*

eTable 7. Absolute Differences and Relative Ratios of Outpatient Medical and Dental Care Use in the Sensitivity Analysis, Excluding Those Who Reported Initiating Public Assistance Due to Chronic Illness or Disease (n = 2,308)

	Medical care		Dental care	
	**Crude model**	**Adjusted model**	**Crude model**	**Adjusted model**
	**AD (95% CI)**	**AD (95% CI) [a]**	**AD (95% CI)**	**AD (95% CI) [a]**
**Expenditures (JPY)**	3,544 (1,795–5,293)	2,856 (1,391–4,322)	2,233 (1,900–2,567)	2,224 (1,893–2,555)
**Visits (times)**	0.47 (0.37–0.58)	0.36 (0.26–0.45)	0.19 (0.16–0.22)	0.19 (0.16–0.22)
**Unit costs (JPY)**	895 (377–1,412)	821 (356–1,287)	1,686 (1,383–1,989)	1,686 (1,382–1,989)
	**RR (95% CI)**	**RR (95% CI) [a]**	**RR (95% CI)**	**RR (95% CI) [a]**
**Expenditures**	1.21 (1.10–1.33)	1.21 (1.10–1.32)	2.26 (2.03–2.51)	2.27 (2.04–2.52)
**Visits**	1.30 (1.23–1.37)	1.28 (1.21–1.35)	1.88 (1.71–2.07)	1.89 (1.71–2.08)
**Unit costs**	1.09 (1.04–1.15)	1.09 (1.04–1.14)	1.53 (1.42–1.64)	1.53 (1.42–1.64)

Abbreviations: AD, absolute difference; RR, relative ratio; CI, Confidence Interval; JPY, Japanese Yen.

[a] Adjusted for age and sex in the month the participants were certified for public assistance and the year when the participants were certified for public assistance.

Expenditures, visits, and unit costs are shown as averages per month.

© 2025 Shiota C et al. *JAMA Health Forum.*

eTable 8. Interrupted-Time Series Analysis of Medical and Dental Care Use in the Sensitivity Analysis, Excluding Those Who Reported Initiating Public Assistance Due to Chronic Illness or Disease (n = 2,308)

Outcome variables	Level change (95%CI)	Trend before public assistance certification (95%CI)	Trend after public assistance certification (95%CI)
**Medical care**			
**Expenditures (JPY)**	909 (-1,384–3,201)	466 (266–666)	-32 (-254–190)
**Visits (times)**	0.24 (0.11–0.37)	0.03 (0.02–0.04)	0.006 (-0.007–0.02)
**Unit cost (JPY)**	952 (307–1,597)	106 (47–165)	-27 (-93–39)
**Dental care**			
**Expenditures (JPY)**	2,339 (1,853–2,826)	13 (-23–48)	-36 (-100–27)
**Visits (times)**	0.21 (0.17–0.26)	-0.00001 (-0.004–0.004)	-0.004 (-0.01–0.001)
**Unit cost (JPY)**	769 (596–941)	11 (-3.3–25)	13 (-10–36)

Abbreviations: CI, Confidence Interval; JPY, Japanese Yen.

This analysis is calculated using the option “family(gaussian)” to converge.

Adjusted for age and sex in the month the participants were certified for public assistance and the year when the participants were certified for public assistance.

Level change refers to the amount of change in the intercept of the outcome from immediately before to immediately after public assistance certification.

Expenditures, visits, and unit costs are shown as averages per month.

© 2025 Shiota C et al. *JAMA Health Forum.*

eTable 9. Descriptive Statistics of Outpatient Medical and Dental Care Use in the Sensitivity Analysis, Excluding Those Who Used the Emergency Department (n = 2,691)

		Public assistance certification				
		Before		After		Price elasticity
		Mean	SD	Mean	SD	
Medical care	Expenditures	16,283	53,892	22,263	54,845	-0.16
	Visits	1.51	2.04	2.03	2.46	-0.15
	Unit costs	10,208	34,751	11,524	33,065	-0.06
Dental care	Expenditures	1,761	3,778	3,944	6,884	-0.38
	Visits	0.22	0.44	0.41	0.66	-0.30
	Unit costs	3,254	4,967	4,819	6,065	-0.19

Abbreviations: SD, standard deviation; JPY, Japanese Yen.

© 2025 Shiota C et al. *JAMA Health Forum.*

eTable 10. Absolute Differences and Relative Ratios of Outpatient Medical and Dental Care Use in the Sensitivity Analysis, Excluding Those Who Used the Emergency Department (n = 2,691)

	Medical care		Dental care	
	Crude model	Adjusted model	Crude model	Adjusted model
	**AD (95% CI)**	**AD (95% CI) [a]**	**AD (95% CI)**	**AD (95% CI) [a]**
**Expenditures (JPY)**	5,981 (4,008–7,953)	5,110 (3,441–6,779)	2,183 (1,879–2,487)	2,183 (1,880–2,486)
**Visits (times)**	0.52 (0.42–0.61)	0.41 (0.32–0.50)	0.19 (0.16–0.22)	0.19 (0.16–0.22)
**Unit costs (JPY)**	1,315 (492–2,139)	1,243 (557–1,929)	1,565 (1,285–1,845)	1,561 (1,280–1,841)
	**RR (95% CI)**	**RR (95% CI) [a]**	**RR (95% CI)**	**RR (95% CI) [a]**
**Expenditures**	1.37 (1.24–1.51)	1.37 (1.25–1.51)	2.24 (2.03–2.47)	2.24 (2.03–2.47)
**Visits**	1.34 (1.27–1.41)	1.32 (1.25–1.39)	1.88 (1.72–2.05)	1.88 (1.72–2.06)
**Unit costs**	1.13 (1.05–1.22)	1.13 (1.06–1.21)	1.48 (1.38–1.58)	1.48 (1.38–1.59)

Abbreviations: AD, absolute difference; RR, relative ratio; CI, Confidence Interval; JPY, Japanese Yen.

[a] Adjusted for age and sex in the month the participants were certified for public assistance and the year when the participants were certified for public assistance.

Expenditures, visits, and unit costs are shown as averages per month.

© 2025 Shiota C et al. *JAMA Health Forum.*

eTable 11. Interrupted-Time Series Analysis of Medical and Dental Care Use in the Sensitivity Analysis, Excluding Those Who Used the Emergency Department (n = 2,691)

Outcome variables	Level change (95% CI)	Trend before public assistance certification (95% CI)	Trend after public assistance certification (95% CI)
**Medical care**			
**Expenditures (JPY)**	2,494 (170–4,820)	582 (388–776)	56 (-212–323)
**Visits (times)**	0.23 (0.11–0.35)	0.04 (0.03–0.05)	0.003 (-0.01–0.01)
**Unit cost (JPY)**	1,210 (98–2,382)	168 (98–238)	7.2 (-82–96)
**Dental care**			
**Expenditures (JPY)**	2,298 (1,840–2,751)	23 (-11–57)	-46 (-106–13)
**Visits (times)**	0.22 (0.17–0.26)	0.001 (-0.003–0.005)	-0.006 (-0.01–0.001)
**Unit cost (JPY)**	761 (602–919)	8.9 (-4.5–22)	12 (-9.8–33)

Abbreviations: CI, Confidence Interval; JPY, Japanese Yen.

Adjusted for age and sex in the month the participants were certified for public assistance and the year when the participants were certified for public assistance.

Level change refers to the amount of change in the intercept of the outcome from immediately before to immediately after public assistance certification.

Expenditures, visits, and unit costs are shown as averages per month.

© 2025 Shiota C et al. *JAMA Health Forum.*

eTable 12. Comparison of Sex and Age Distribution Between the Target Population in This Study and the Public Assistance Recipients Based on National Data

	Target Population in This Study	Public Assistance Recipients Based on National Data
**Sex**		
**Male**	48.1%	49.6%
**Female**	51.9%	50.4%
**Age**		
**0í 19**	15.2%	8.6%
**20–29**	6.0%	2.8%
**30–39**	10.7%	4.7%
**40–49**	13.4%	8.9%
**50–59**	17.9%	14.4%
**60–64**	10.5%	7.9%
**65–69**	10.0%	9.1%
**70–74**	16.3%	43.6%
**Total**	100.0%	100.0%

© 2025 Shiota C et al. *JAMA Health Forum.*

eFigure 1. Design of the Analytic Sample (n = 2,893)

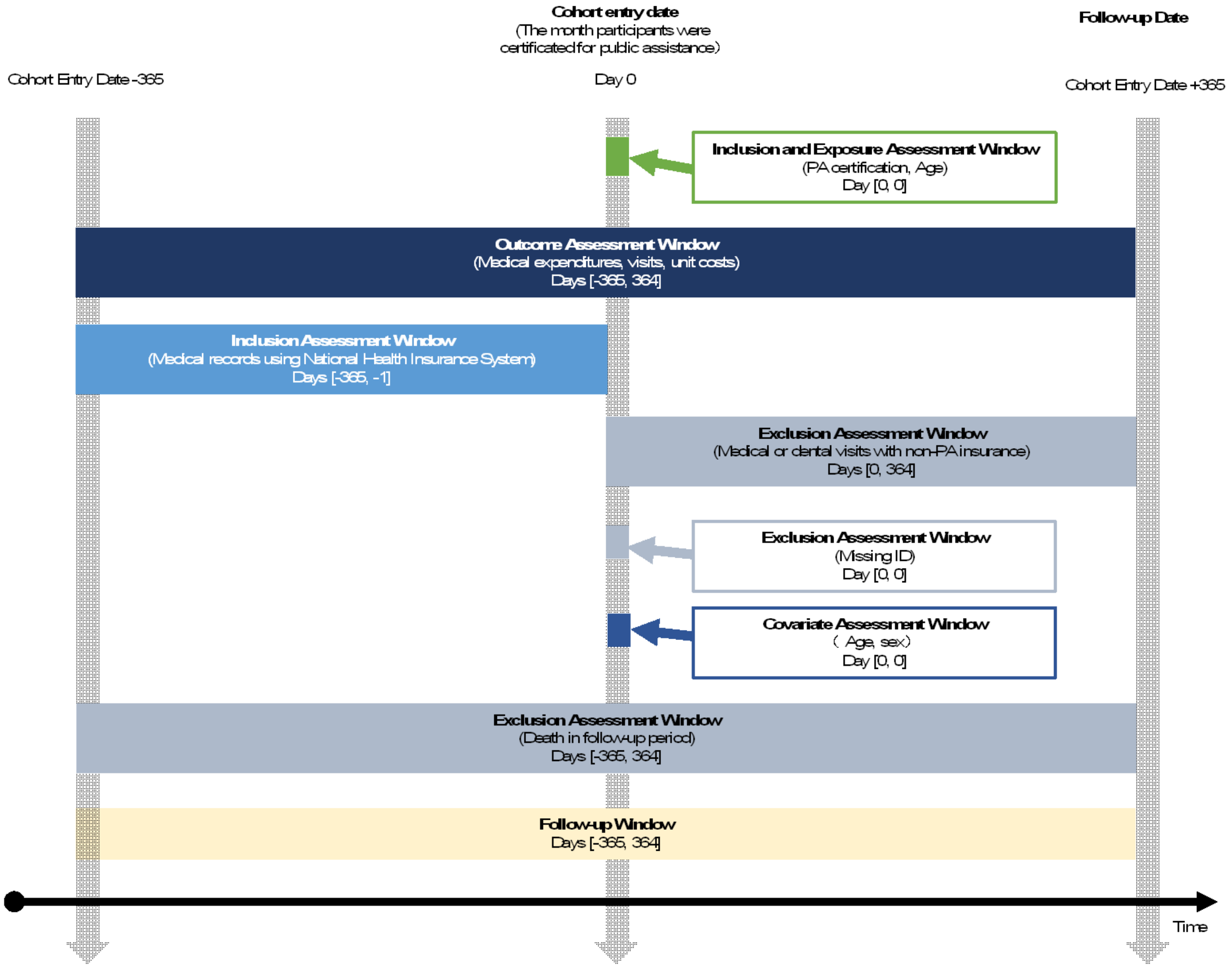

Abbreviations: PA, public assistance.

© 2025 Shiota C et al. *JAMA Health Forum.*

eFigure 2. Participant Flow for the Analytic Sample (n =2,893)

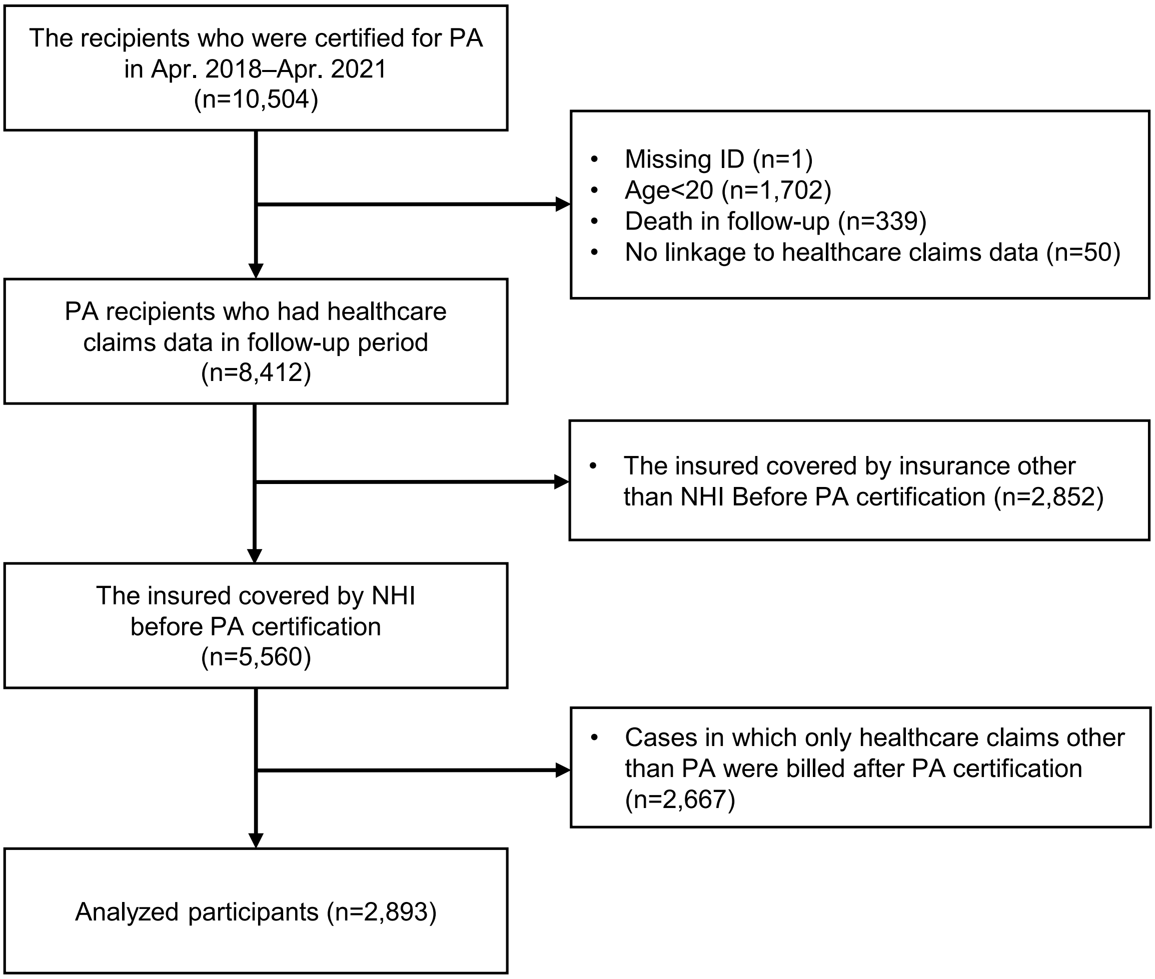

Abbreviations: PA, public assistance; NHI, National Health Insurance.

© 2025 Shiota C et al. *JAMA Health Forum.*

eFigure 3. Design of the Analytic Sample in the Sensitivity Analysis, Excluding Those Whose Healthcare Expenditures were Zero After Public Assistance Certification (n = 2,821)

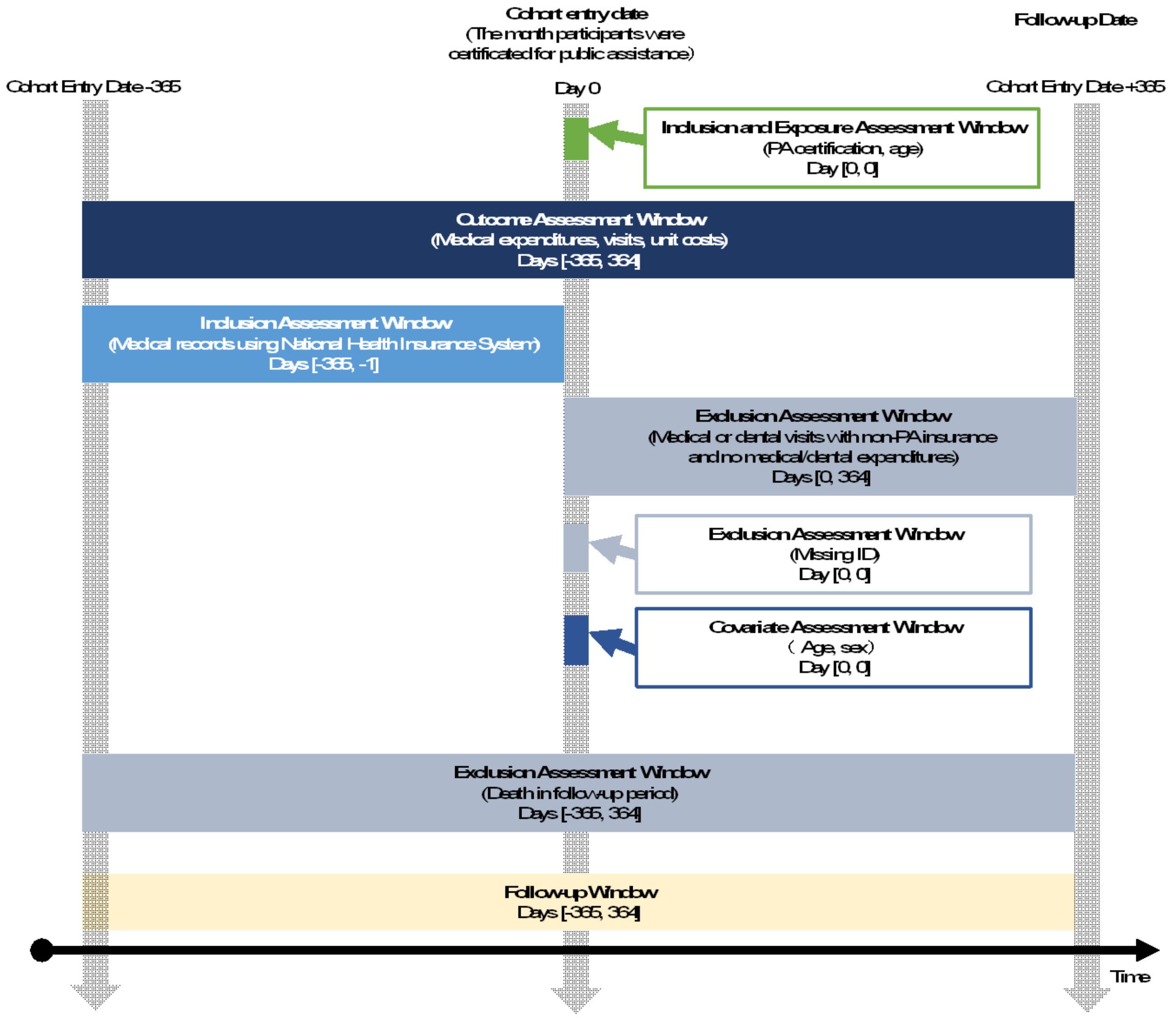

Abbreviations: PA, public assistance.

© 2025 Shiota C et al. *JAMA Health Forum.*

eFigure 4. Participant Flow for the Analytic Sample in the Sensitivity Analysis, Excluding Those with Zero Healthcare Expenditures After Public Assistance Certification (n = 2,821)

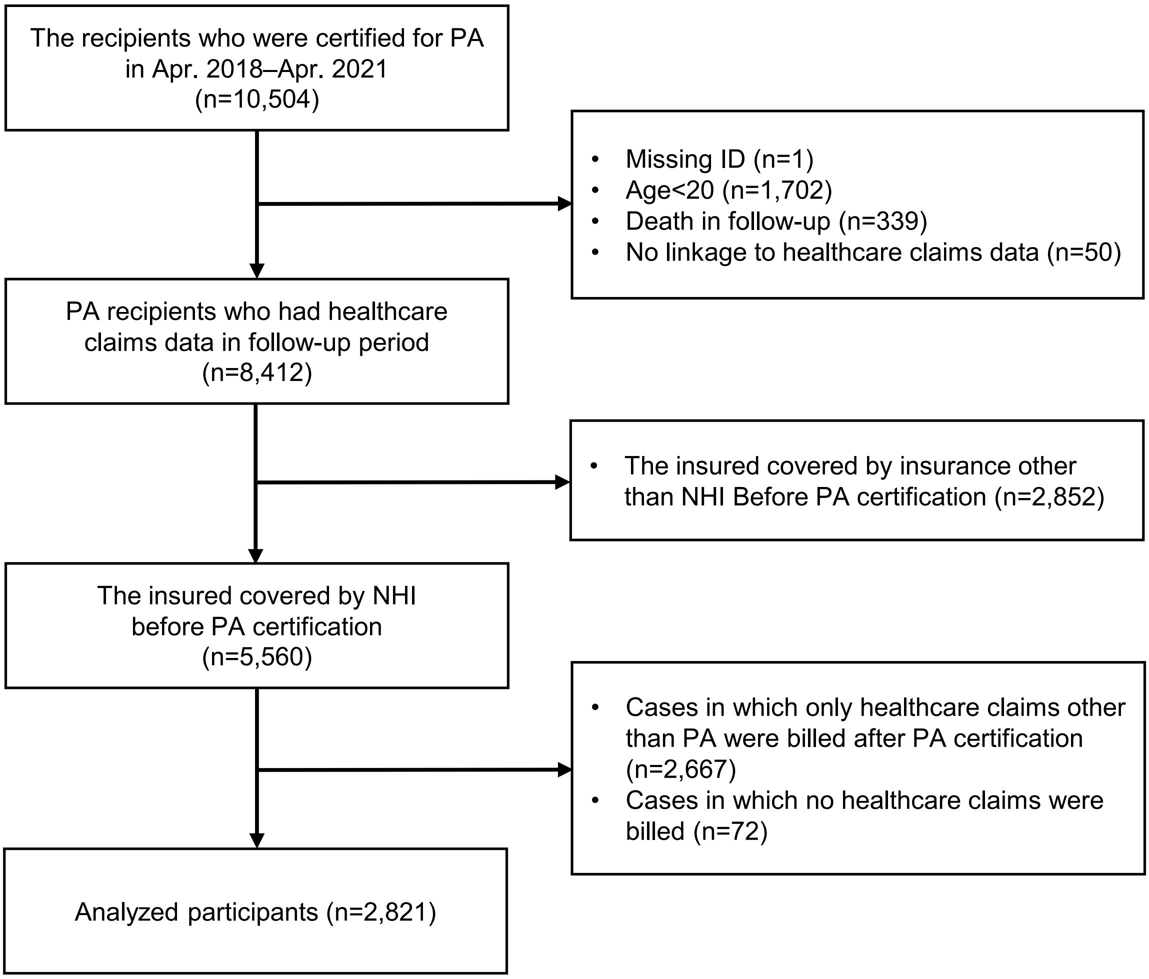

Abbreviations: PA, public assistance; NHI. National Health Insurance

© 2025 Shiota C et al. *JAMA Health Forum.*

eFigure 5. Interrupted Time-Series Analysis of Outpatient Medical Care Use in the Sensitivity Analysis, Excluding Those with Zero Healthcare Expenditures After Public Assistance Certification (n = 2,821)

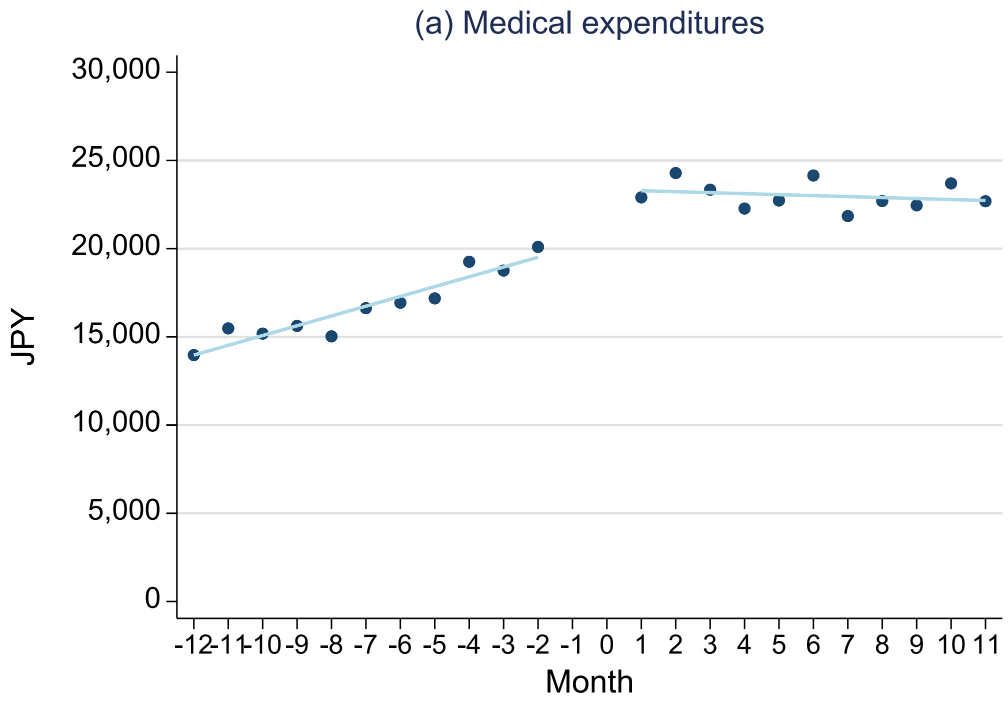

Level change (95%CI): 3,217 JPY (865–5,569); Trend before public assistance certification (95%CI): 555 JPY (356–754); Trend after public assistance certification (95%CI): -56 JPY (-304–193).

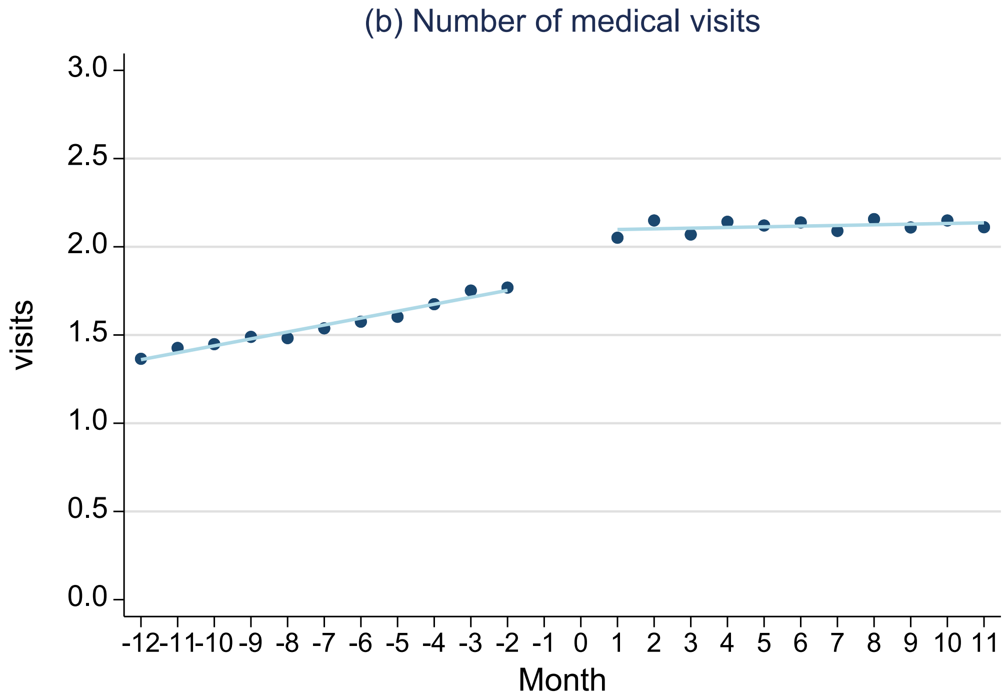

Level change (95%CI): 0.31 visits (0.19–0.43); Trend before public assistance certification (95%CI): 0.04 visits (0.03–0.05); Trend after public assistance certification (95%CI): 0.004 visits (-0.009–0.016).

© 2025 Shiota C et al. *JAMA Health Forum.*

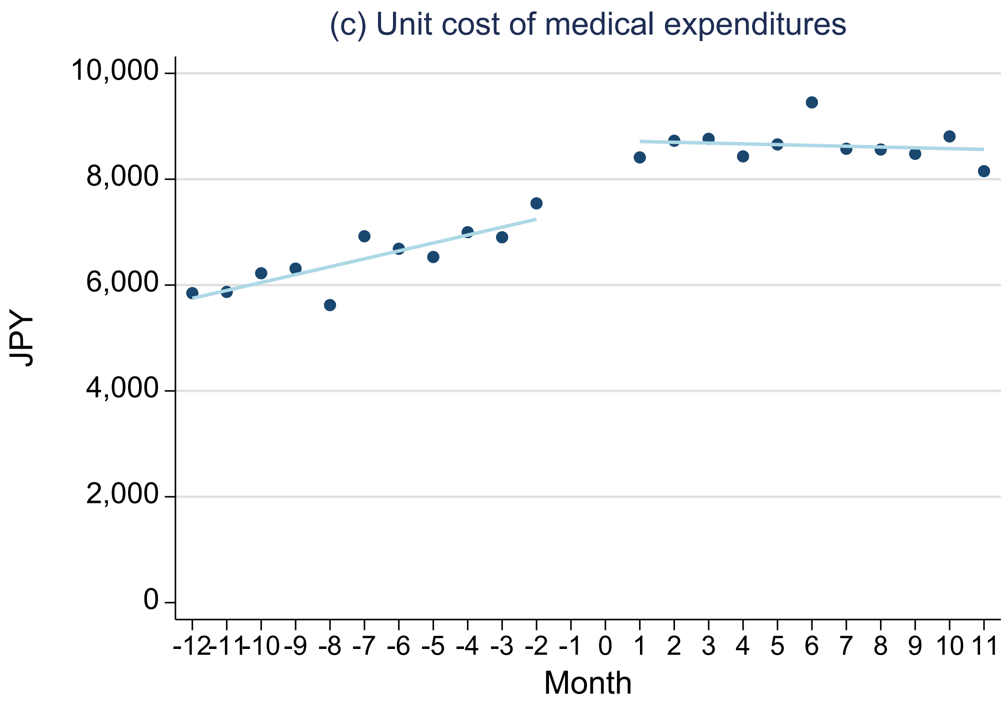

Level change (95%CI): 1,320 JPY (525–2,115); Trend before public assistance certification (95%CI): 149 JPY (72–227);

Trend after public assistance certification (95%CI): -15 JPY (-86–56).

Abbreviations: PA, public assistance; JPY, Japanese Yen; CI, confidence interval.

The month of the PA certification was defined as "month 0." The 12 months prior were identified as months -12 to -1, whereas the 12 months following the PA certification were defined as months 0 to 11.

© 2025 Shiota C et al. *JAMA Health Forum.*

eFigure 6. Interrupted Time-Series Analysis of Outpatient Dental Care Use in the Sensitivity Analysis, Excluding Those with Zero Healthcare Expenditures After Public Assistance Certification (n = 2,821)

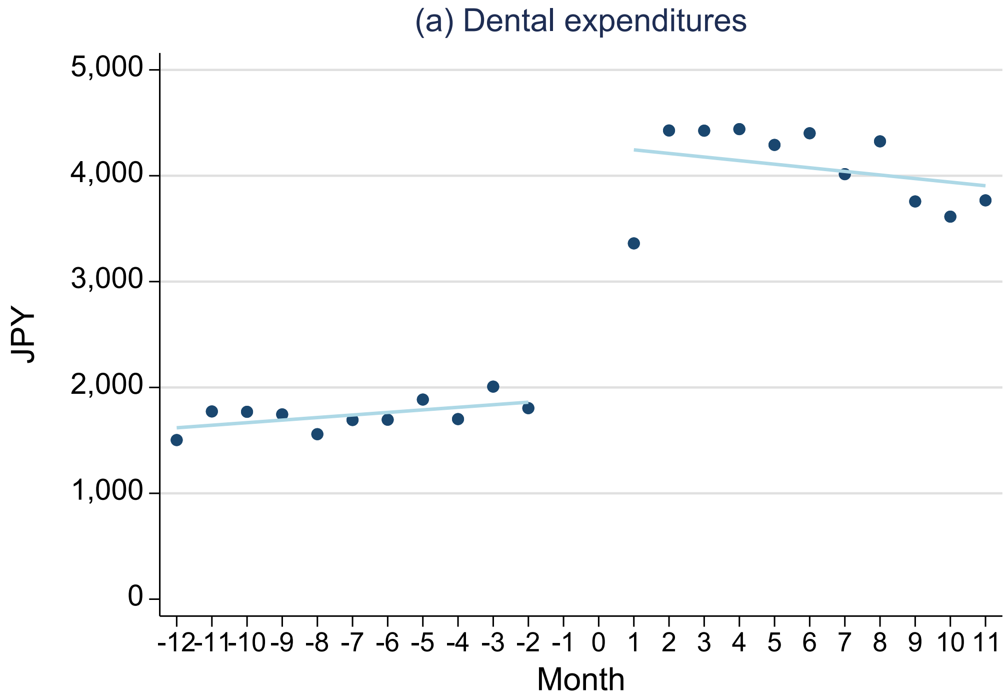

Level change (95%CI): 2,360 JPY (1,920–2,800); Trend before public assistance certification (95%CI):24 JPY (-8.6–57);

Trend after public assistance certification (95%CI): -34 JPY (-93–25).

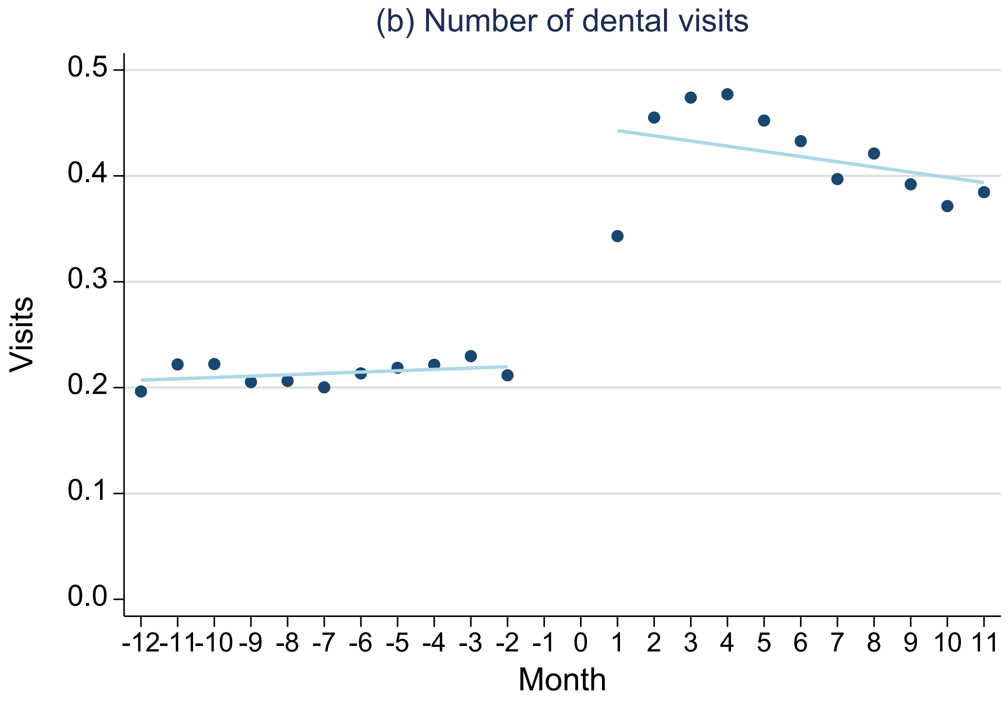

Level change (95%CI):0.22 visits (0.18–0.26); Trend before public assistance certification (95%CI):0.001 visits (-0.002–0.005);

Trend after public assistance certification (95%CI): -0.005 visits (-0.01–0.0002).

© 2025 Shiota C et al. *JAMA Health Forum.*

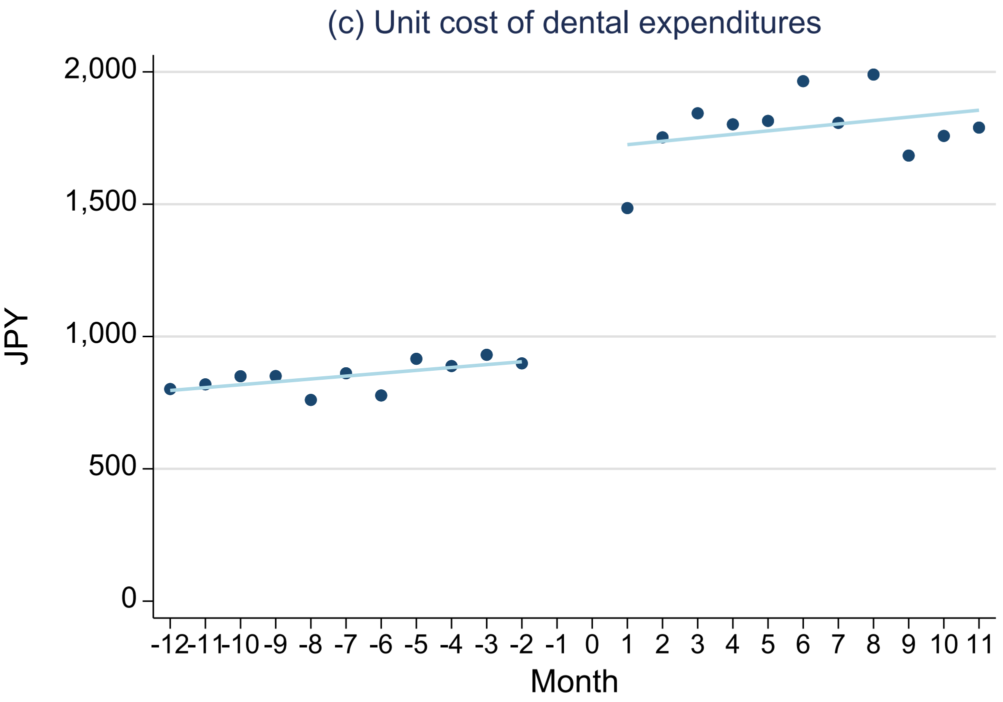

Level change (95%CI): 810 JPY (654–965); Trend before public assistance certification (95%CI): 11 JPY (-2.0–24); Trend after public assistance certification (95%CI): 13 JPY (-8.2–34).

Abbreviations: PA, public assistance; JPY, Japanese Yen; CI, confidence interval.

The month of the PA certification was defined as "month 0." The 12 months prior were identified as months -12 to -1, whereas the 12 months following the PA certification were defined as months 0 to 11.

© 2025 Shiota C et al. *JAMA Health Forum.*

eFigure 7. Interrupted Time-Series Analysis of Outpatient Medical Care Use in the Sensitivity Analysis, Excluding Those Who Reported Initiating Public Assistance Due to Chronic Illness or Disease (n = 2,308)

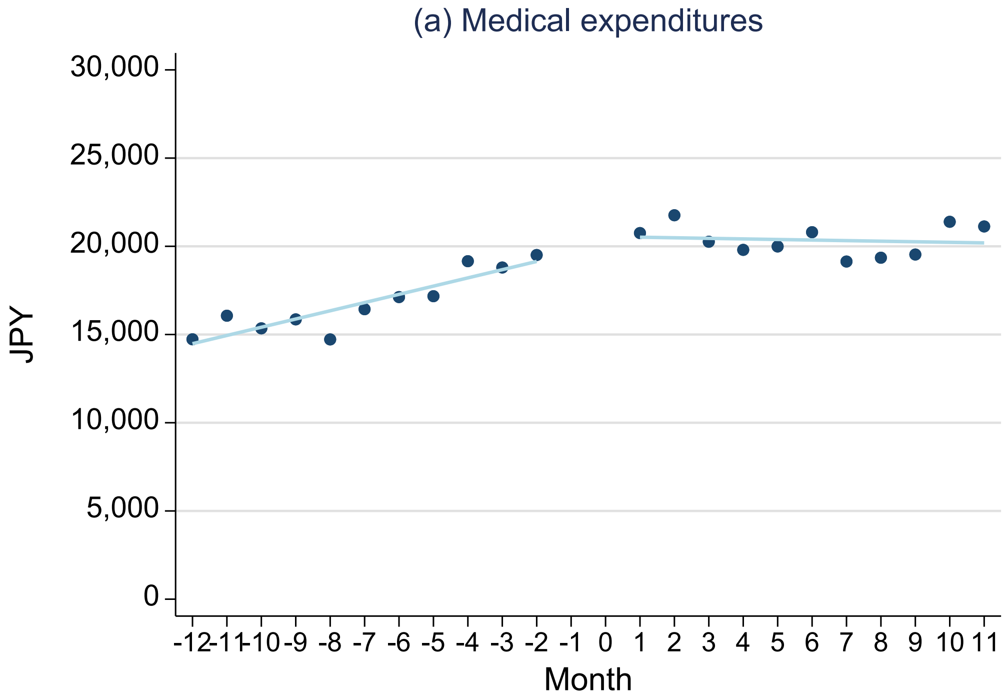

Level change (95%CI): 909 JPY (-1,384–3,201); Trend before public assistance certification (95%CI): 466 JPY (266–666);
Trend after public assistance certification (95%CI): -32 JPY (-254–190).

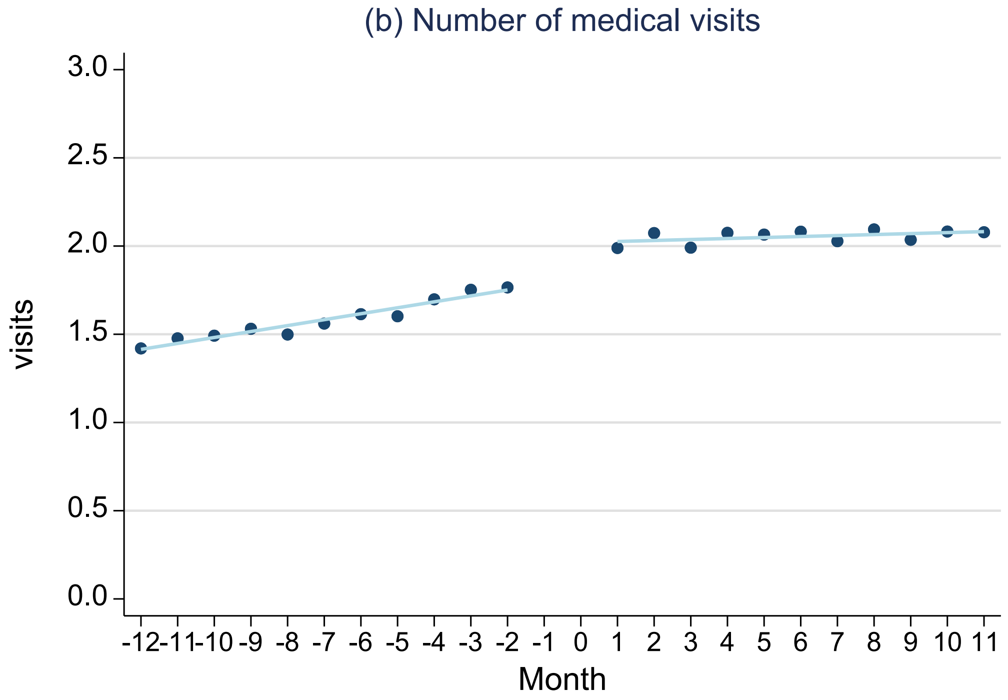

Level change (95%CI):0.24 visits (0.11–0.37); Trend before public assistance certification (95%CI):0.03 visits (0.02–0.04);
Trend after public assistance certification (95%CI): 0.006 visits (-0.007–0.02).

© 2025 Shiota C et al. *JAMA Health Forum.*

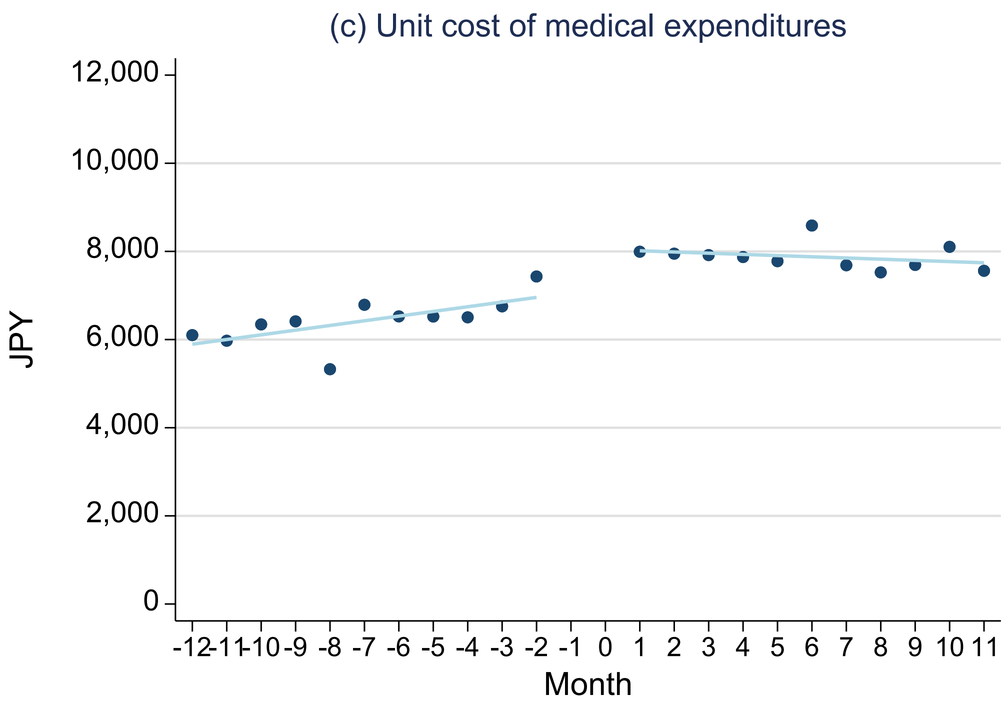

Level change (95%CI): 952 JPY (307–1,597); Trend before public assistance certification (95%CI): 106 JPY (47–165);
Trend after public assistance certification (95%CI): -27 JPY (-93–39).

Abbreviations: PA, public assistance; JPY, Japanese Yen; CI, confidence interval.

The month of the PA certification was defined as "month 0." The 12 months prior were identified as months -12 to -1, whereas the 12 months following the PA certification were defined as months 0 to 11.

© 2025 Shiota C et al. *JAMA Health Forum.*

eFigure 8. Interrupted Time-Series Analysis of Outpatient Dental Care Use in the Sensitivity Analysis, Excluding Those Who Reported Initiating Public Assistance Due to Chronic Illness or Disease (n = 2,308)

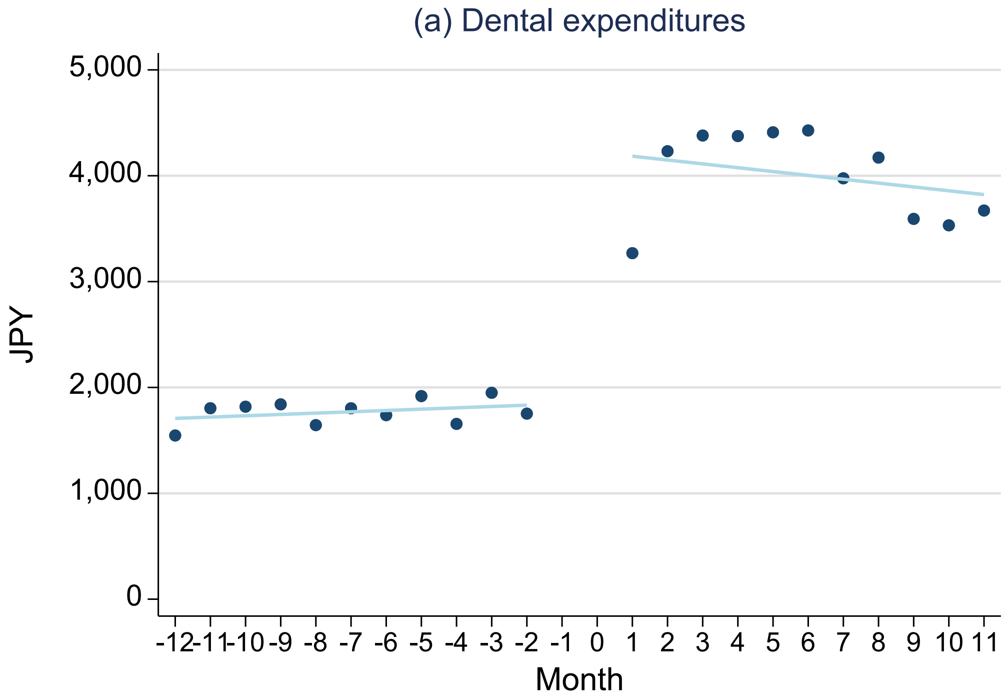

Level change (95%CI):2,339 JPY (1,853–2,826); Trend before public assistance certification (95%CI):13 JPY (-23–48); Trend after public assistance certification (95%CI): -36 JPY (-100–27).

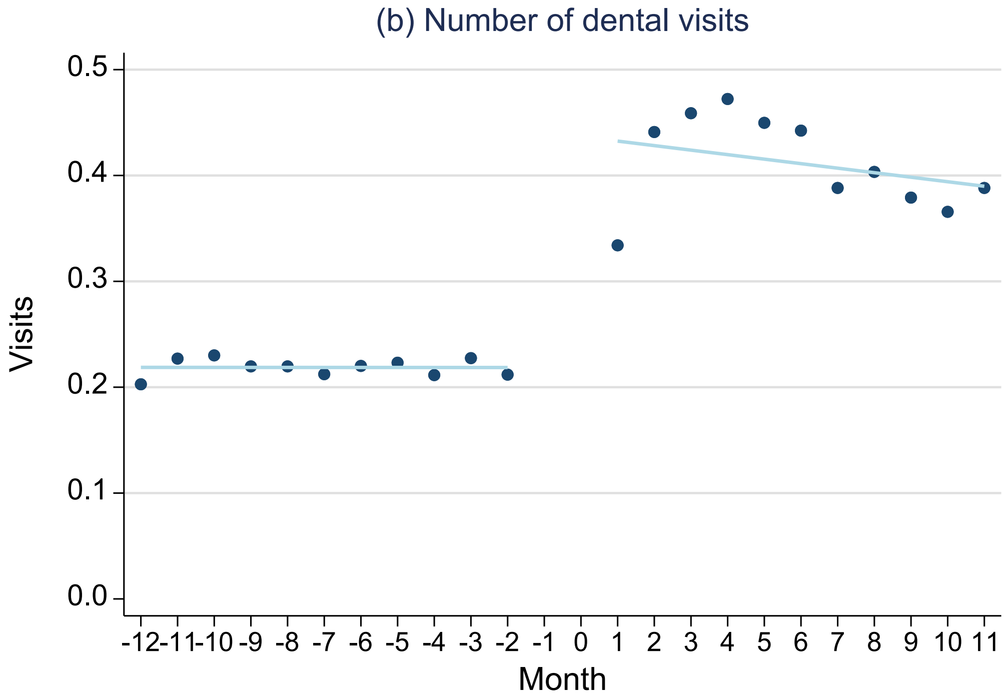

Level change (95%CI):0.21 visits (0.17–0.26); Trend before public assistance certification (95%CI): -0.00001 visits (-0.004–0.004); Trend after public assistance certification (95%CI): -0.004 visits (-0.01–0.001).

© 2025 Shiota C et al. *JAMA Health Forum.*

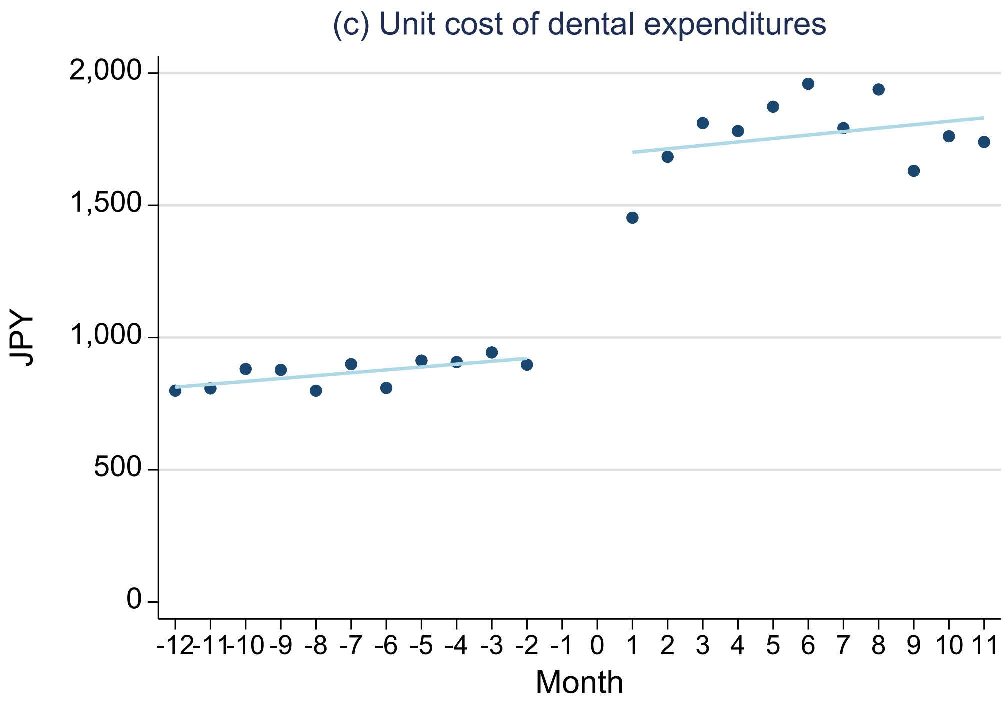

Level change (95%CI):769 JPY (596–941); Trend before public assistance certification (95%CI): 11 JPY (-3.3–25);
Trend after public assistance certification (95%CI): 13 JPY (-10–36).

Abbreviations: PA, public assistance; JPY, Japanese Yen; CI, confidence interval.

The month of the PA certification was defined as "month 0." The 12 months prior were identified as months -12 to -1, whereas the 12 months following the PA certification were defined as months 0 to 11.

© 2025 Shiota C et al. *JAMA Health Forum.*

eFigure 9. Interrupted Time-Series Analysis of Outpatient Medical Care Use in the Sensitivity Analysis, Excluding Those Who Used the Emergency Department (n = 2,691)

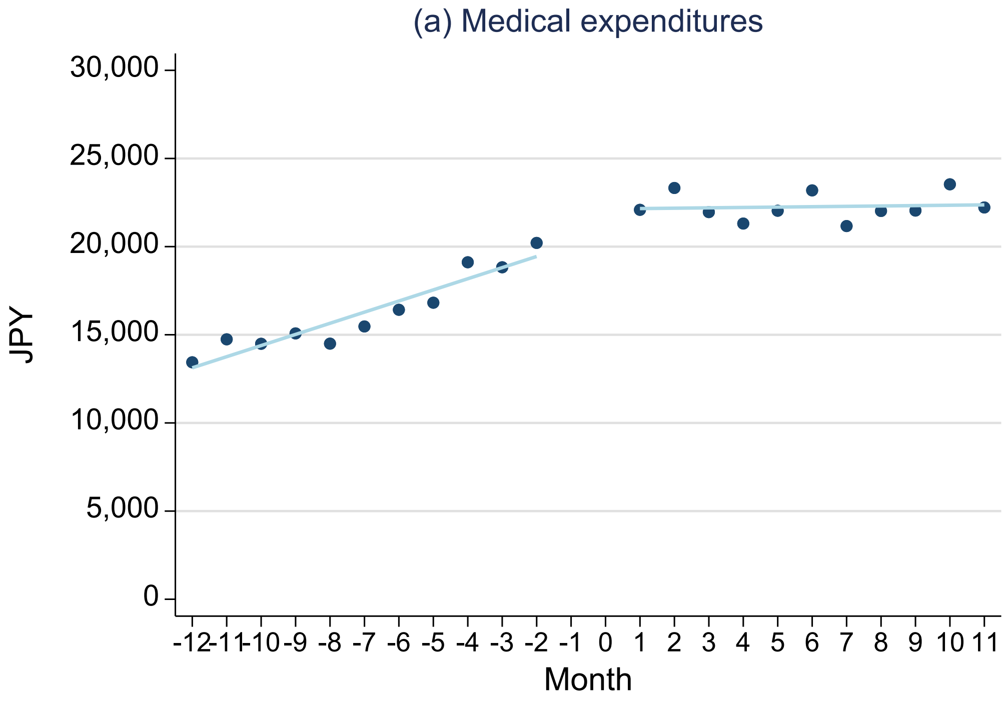

Level change (95%CI): 2,494 JPY (170–4,820); Trend before public assistance certification (95%CI):582 JPY (388–776); Trend after public assistance certification (95%CI): 56 JPY (-212–323).

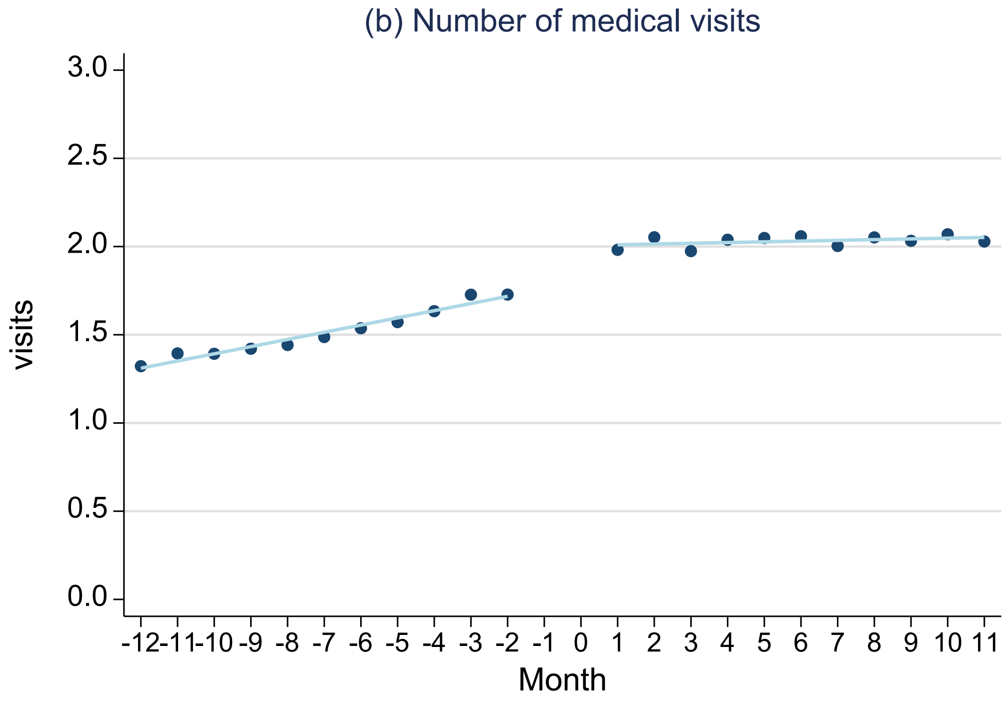

Level change (95%CI): 0.23 visits (0.11–0.35); Trend before public assistance certification (95%CI): 0.04 visits (0.03–0.05); Trend after public assistance certification (95%CI): 0.003 visits (-0.01–0.01).

© 2025 Shiota C et al. *JAMA Health Forum.*

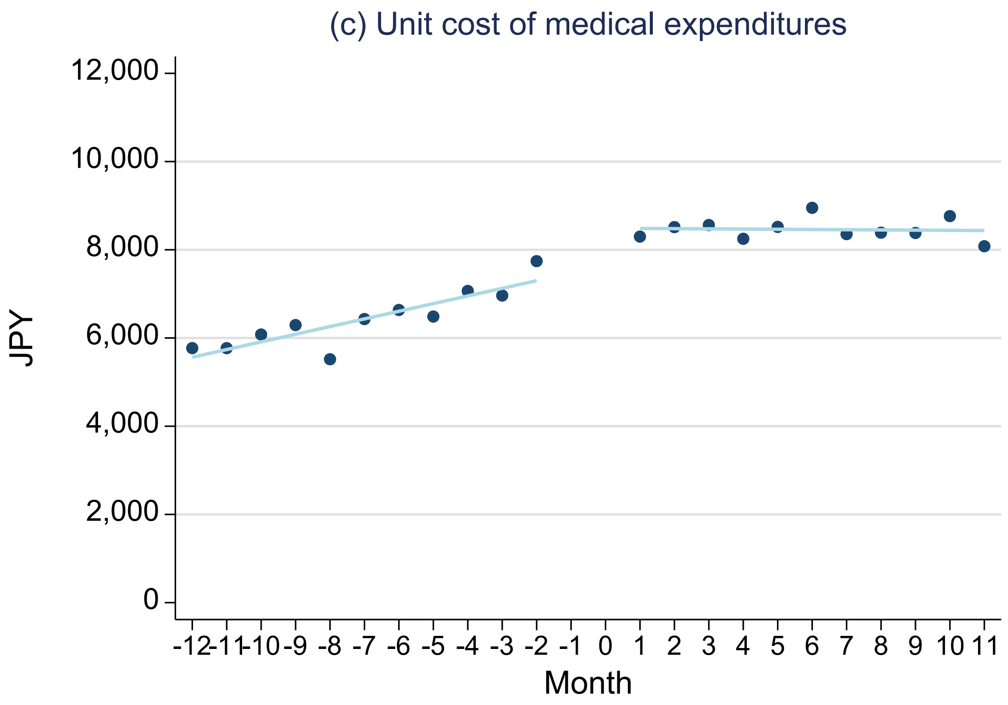

Level change (95%CI): 1,210 JPY (98–2,382); Trend before public assistance certification (95%CI):23 JPY (-11–57);
Trend after public assistance certification (95%CI): -46 JPY (-106–13).

Abbreviations: PA, public assistance; JPY, Japanese Yen; CI, confidence interval.

The month of the PA certification was defined as "month 0." The 12 months prior were identified as months -12 to -1, whereas the 12 months following the PA certification were defined as months 0 to 11.

© 2025 Shiota C et al. *JAMA Health Forum.*

eFigure 10. Interrupted Time-Series Analysis of Outpatient Dental Care Use in the Sensitivity Analysis, Excluding Those Who Used the Emergency Department (n = 2,691)

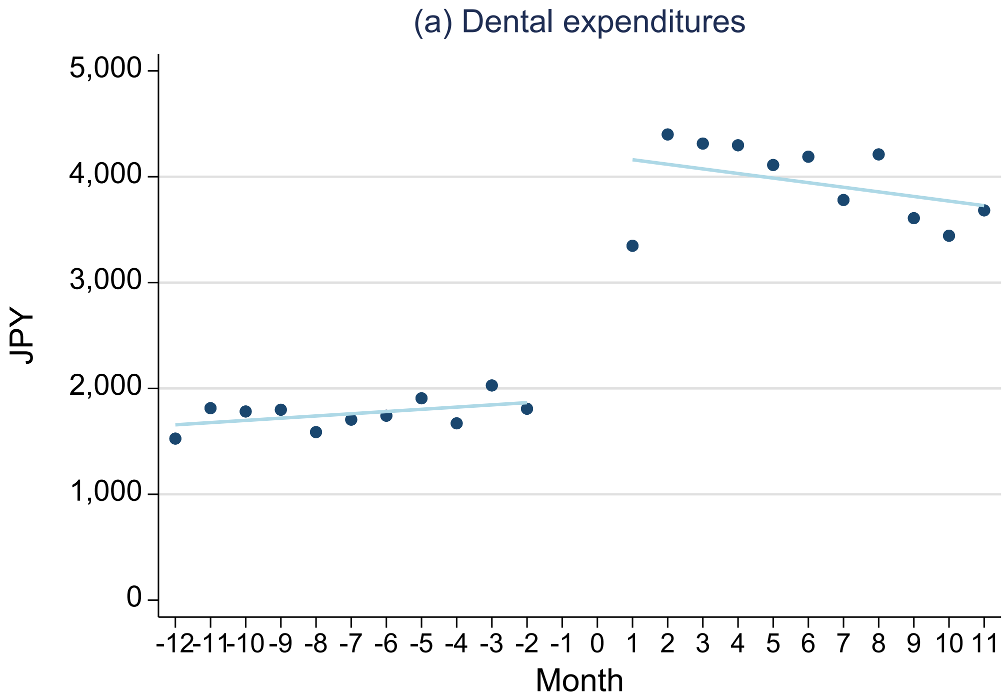

Level change (95%CI): 2,298 JPY (1,840–2,751); Trend before public assistance certification (95%CI):23 JPY (-11–57); Trend after public assistance certification (95%CI): -46 JPY (-106–13).

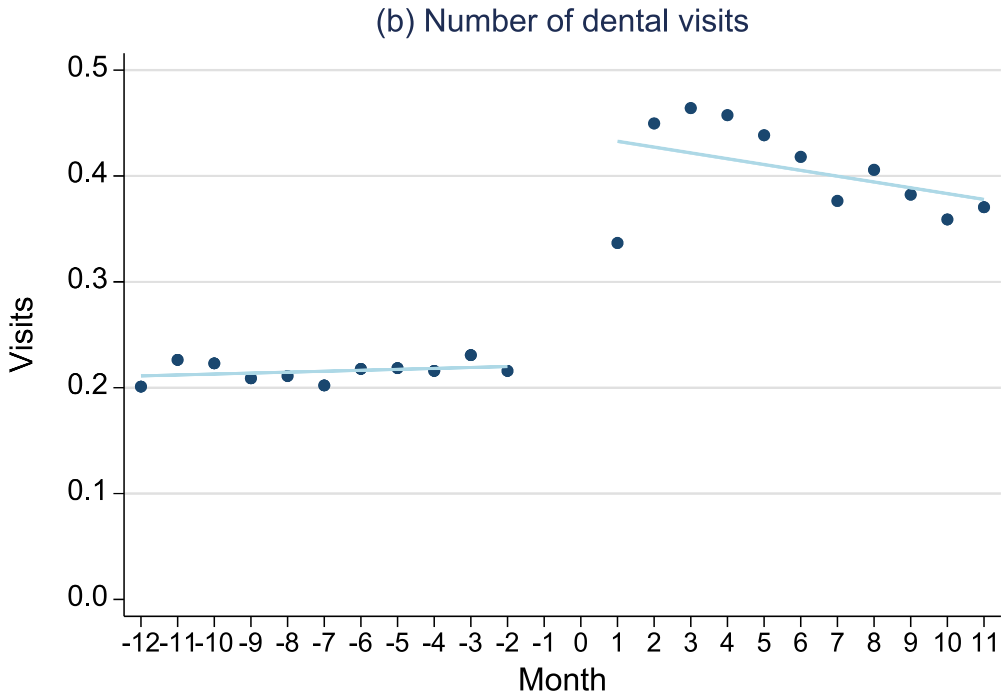

Level change (95%CI): 0.22 visits (0.17–0.26); Trend before public assistance certification (95%CI):0.001 visits (-0.003–0.005); Trend after public assistance certification (95%CI): -0.006 visits (-0.01–0.001).

© 2025 Shiota C et al. *JAMA Health Forum.*

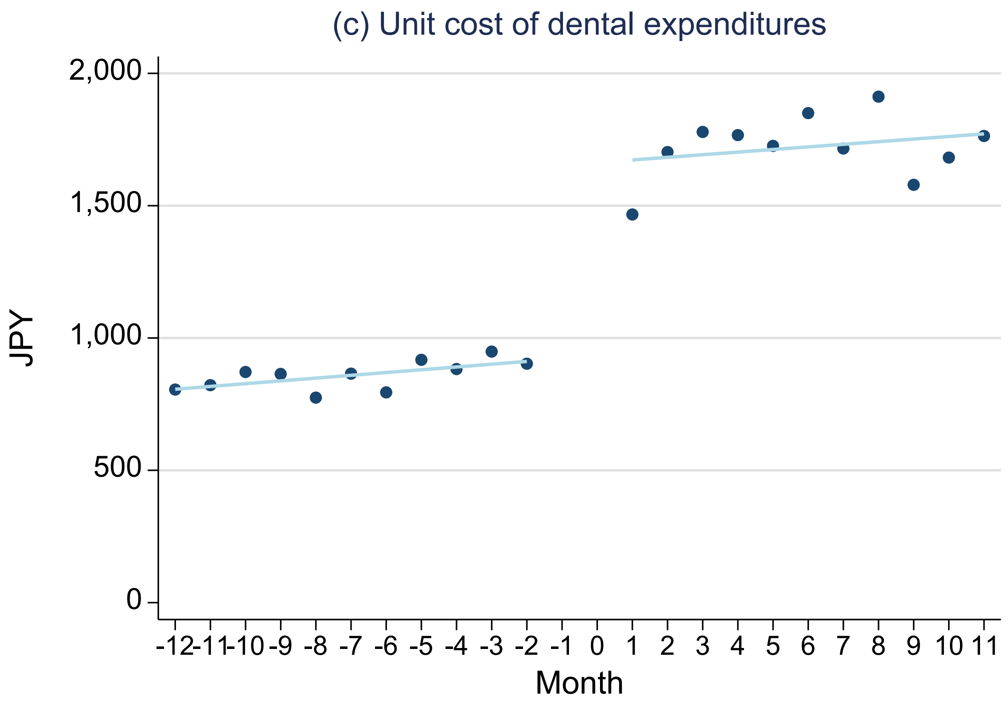

Level change (95%CI): 761 JPY (602–919); Trend before public assistance certification (95%CI):8.9 JPY (-4.5–22);
Trend after public assistance certification (95%CI): 12 JPY (-9.8–33).

Abbreviations: PA, public assistance; JPY, Japanese Yen; CI, confidence interval.

The month of the PA certification was defined as "month 0." The 12 months prior were identified as months -12 to -1, whereas the 12 months following the PA certification were defined as months 0 to 11.

© 2025 Shiota C et al. *JAMA Health Forum.*

eMethods

eMethods 1. Introduction to the Japanese Healthcare System

In Japan, people are mandated to enroll in one of the three health insurance plans—NHI, Employees' Health Insurance, or the Latter-Stage Older Persons Health Care System—based on their social and demographic status. Co-payment rates typically range from 10% to 30%, and most treatments are covered, excluding maternity costs, medical examinations, and preventive services.

eMethods 2. Formulas in Generalized Estimating Equation Models and Interrupted Time-Series Analysis Models Used in This Study

ITSA models using the gamma family and identity link function were implemented via the xtitsa command, which wraps GEE modeling while accounting for autocorrelation.[3] In the ITSA model, comparing individuals themselves before and after PA certification can reduce confounding from fixed individual-level health characteristics. Accordingly, the GEE models, we used the following formulas in Stata to calculate relative ratios:

xtset "ID"

xtgee "outcome variable (such as monthly expenditure)" i."flag variable defining before and after PA certification" i.sex i.age variable, family(gamma) link(identity)

Additionally,

xtset "ID"

xtgee "outcome variable (such as monthly expenditure)" i."flag variable (before and after)" i.sex i.age variable, family(gamma) link(log) eform

was applied to calculate absolute differences.

In the ITSA models, we used the following formulas in Stata to calculate level change and trends:

xtitsa "outcome variable (monthly expenditure)" i.sex i.age variable, single trperiod family(gamma) link(identity) vce(r) posttrend figure replace

© 2025 Shiota C et al. *JAMA Health Forum.*

eMethods 3. Overview of Three Sensitivity Analyses

Three patterns of sensitivity analyses were also conducted. First, to address potential withdrawal from the PA, we limited our sample to individuals with at least one outpatient claim documented under the PA receipt type during the follow-up period. This enabled us to concentrate on participants who were likely to have remained enrolled in the PA. Second, we analyzed participants, excluding those who reported initiating PA due to chronic illness or disease. This aimed to address two potential bidirectional causal relationships: cost sharing may decrease after PA certification, leading to increased health service use, or deteriorating health may reduce income, prompting enrollment in PA with zero cost sharing. Third, we conducted a sensitivity analysis excluding individuals who used emergency outpatient services during follow-up, given motivations for emergency visits differ substantially from general outpatient care and may respond differently to cost-sharing changes.

eMethods 4. Formula for Calculating Price Elasticities of Expenditure, Number of Visits, and Unit Cost

Price elasticity in the context of a change in the copayment rate to zero has been described in a previous study.[4] The calculation for arc price elasticity is as follows:

$$\text{Arc price elasticity} = \frac{Q2\text{-}Q1}{(Q2+Q1)/2} \Big/ \frac{P2\text{-}P1}{(P2+P1)/2} = (Q2\text{-}Q1)/(Q2+Q1)$$

Where P1 and P2 are the copayment rates before and after the intervention (30% to 0%), respectively, and Q1 and Q2 represent the healthcare expenditure before and after the intervention, respectively. Given that the copayment rate has changed from 30% to 0%, the formula is simplified to (Q2-Q1)/(Q2+Q1). We applied this formula to calculate the price elasticities for each outcome indicator.

© 2025 Shiota C et al. *JAMA Health Forum.*

eResults

eResults 1: Results of three sensitivity analyses

In the first sensitivity analyses (eFigure 3), we analyzed the data of 2,821 participants who had outpatient medical or dental records identified under the PA system. Patient characteristics are detailed in eTable 2. Descriptive statistics and price elasticities were similar to those in the primary analysis (eTable 3). Results from both the GEE models and ITSA (eTables 4 and 5) confirmed consistent trends and level changes across all outcomes, reinforcing the robustness of the primary findings.

Additional sensitivity analyses were conducted. One excluded participants who reported initiating PA due to chronic illness or disease (eTables 7 and 8; eFigures 7 and 8), while another excluded those who used emergency department services during the follow-up period (eTables 10 and 11; eFigures 9 and 10). Both analyses yielded results consistent with the main analysis.

eReferences:

1. Ministry of Health, Labour and Welfare. Basic Data on Medical Insurance. Published online December 2024. https://www.mhlw.go.jp/content/kiso_r04.pdf

2. Ministry of Health, Labour and Welfare. National Survey on Public Assistance Recipients 2022. Published online March 22, 2024. https://www.e-stat.go.jp/stat-search/file-download?statInfId=000040164402&fileKind=2

3. Linden A. XTITSA: Stata module for performing interrupted time-series analysis for panel data. *Statistical Software Components*. Published online March 23, 2021. Accessed February 26, 2024. https://ideas.repec.org/c/boc/bocode/s458903.html

4. Fukuma S, Kato H, Takaku R, Tsugawa Y. Effect of no cost sharing for paediatric care on healthcare usage by household income levels: regression discontinuity design. *BMJ Open*. 2023;13(8):e071976.

© 2025 Shiota C et al. *JAMA Health Forum.*
