# Supplementary material for: No Cost Sharing for Public Assistance Recipients and Health Service Usage in Japan
Source: JAMA Health Forum. 2025 Oct 24;6(10):e253713. doi: 10.1001/jamahealthforum.2025.3713 (PMC12552928; doi:10.1001/jamahealthforum.2025.3713)
Supplement: Supplement 2. — Data Sharing Statement [file jamahealthforum-e253713-s002.pdf]

Data Sharing Statement

Shiota. No Cost Sharing for Public Assistance Recipients and Health Service Usage in Japan. *JAMA Health Forum*. Published October 24, 2025. doi:10.1001/jamahealthforum.2025.3713

Data
Data available: No

Additional Information
Explanation for why data not available: We used restricted data from a municipality which has the National Health Insurance, the National Survey on Public Assistance Recipients and Basic Resident Register and per our Data Use agreement we can not share individual data.
